# Supplementary material for: Super-pangenome analyses highlight genomic diversity and structural variation across wild and cultivated tomato species
Source: Nat Genet. 2023 Apr 6;55(5):852–60. doi: 10.1038/s41588-023-01340-y (PMC10181942; doi:10.1038/s41588-023-01340-y)
Supplement: Supplementary file 1 — Supplementary Note and Figs. 1–32. [file 41588_2023_1340_MOESM1_ESM.pdf]

nature genetics

Article https://doi.org/10.1038/s41588-023-01340-y

Super-pangenome analyses highlight genomic diversity and structural variation across wild and cultivated tomato species

In the format provided by the
authors and unedited

Supplementary Note 1: Material and methods 2

1.1 Library construction and sequencing 2
1.2 Comparison between the super-pangenome and the previously reported pan-genome 2
1.3 Features of the 9,320 genes exclusively identified in the super-pangenome 2
1.4 Integration of SVs reported in the previous reported pan-SV map 3

Supplementary Note 2: Genome assembly of the 11 tomato accessions 4

Supplementary Note 3: Comparison between the tomato super-pangenome and the previously reported tomato pan-genome 5

Supplementary Note 4: Features and examples of small genetic variants among wild and cultivated tomatoes 6

Supplementary Note 5: SVs that are only present in the tomato super-pangenome 7

Supplementary Figures 8

Supplementary References 41

Supplementary Note 1: Material and methods

1.1 Library construction and sequencing

PacBio SMRT libraries (20-kb fragment size) were constructed using the high molecular weight DNA, based on the protocol of SMRT bell library construction (https://www.pacb.com/support/documentation/), and then sequenced on the PacBio Sequel platform. For short-read sequencing, genomic DNA were fragmented into ~450 bp for paired-end library construction, following manufacturer's instruction (Illumina Inc.). Hi-C sequencing libraries were constructed, according to the proximal Hi-C library protocol. Total RNA were extracted from whole plants using Trizol (Invitrogen CarIsbad, CA, USA), and RNA sequencing libraries were constructed using TruSeqTM RNA sample Prep Kit (Illumina, CA, USA). All abovementioned short-read sequencing libraries were sequenced on the Illumina NovaSeq 6000 platform.

1.2 Comparison between the super-pangenome and the previously reported pan-genome

Regarding the comparison between the super-pangenome constructed in this study and the pan-genome reported in Gao *et al.*[1], amino acid sequences of genes from the *S. galapagense* genomes were extracted as the representative for each core-gene family, and they were aligned to those reported core genes using BLASTP[2], with an E-value threshold of 1×10^{-5}. A gene was regarded as "shared" if presenting both over 90% alignment identity and coverage. To identify genes that were uniquely present in our super-pangenome, we extracted coding sequences of each predicted genes in the 13 tomato genomes and aligned them against the reported tomato pan-genome sequences[1] using BLASTN, with default parameters. Those genes showing alignment identity < 90% or coverage < 75% were retrieved. Transposable elements were then identified from those genes using EDTA (v1.9.4)[3] with parameters "--anno 1 --step all". Genes whose CDS harboring > 25% of transposable elements were excluded, followed by removal of redundancy using Cd-hit[4] (v4.8.1, default parameters), which led to a non-redundant catalog of 9,320 genes.

1.3 Features of the 9,320 genes exclusively identified in the super-pangenome

Genome-wide synteny between the tomato pan-genome reported in Gao *et al.*[1] and each of the 12 tomato accessions used in this study was inferred by GeneTribe (v1.2.0)[5] with default parameters. Among the 9,320 genes, those without syntenic pairs in the tomato pan-genome reported in Gao *et al.*[1] were considered to be arose from non-collinear segments, whereas those with syntenic pairs were regarded as additional alleles of existing genes. To investigate genome-wide distribution of the 9,320 genes, we performed gene synteny inference using GeneTribe between the genome of each of the 12 tomatoes and the *S. galapagens* genome and converted their genomic coordinates to the *S. galapagens* genome. For each of the 9,320 genes, the relative genomic coordinate was determined if it had a matched gene within the synteny block in the *S. galapagens* genome. When the gene synteny was lost, the genomic coordinate of the closest *S. galapagens* gene retaining the synteny was used.

To determine whether the 9,320 genes could be found in tomato populations, we collected

previously released resequencing reads of 321 tomato accessions[6] and mapped them to CDS of the 9,320 genes using BWA mem[7] (version 0.7.10-r789, default parameters). Supplementary alignments and those showing signals of PCR duplicates were subsequently removed using SAMtools (v1.3)[8] view with parameters "-F 512 -F 1024 -F 2048". We next computed alignment coverage of each nucleotide in a given gene (only mapped bases with coverage ≥ 2, base quality ≥ 30 and mapping quality ≥ 30 were considered) using SAMtools depth with parameters "-aa -q 30 -Q 30". If the alignment coverage ≥ 90%, this gene was regarded as "presence" in the given tomato accession.

1.4 Integration of SVs reported in the previous reported pan-SV map

The coordinate of our SV callset was based on the genome of *S. galapagense* LA0436, which is different from that reported in Alonge *et al.* (*S. lycopersicum* cv. Heinz 1706)[9]. To facilitate downstream analyses, we transformed the Heinz 1706-based SV coordinates to LA0436-based one by aligning both 200-bp left and right flanking sequences of each SV breakpoint extracted from the genome of Heinz 1706 to the LA0436 genome using nucmer, setting an alignment threshold of 95% identity and 300-bp match. A total of 163,703 out of 231,962 SVs could be properly lifted over to the LA0436-based coordinate system, the remaining of which consisted of 47,944 SVs that failed to meet the alignment threshold, 8,389 that could not be aligned to the LA0436 genome and 11,926 localized at regions with structural differences between LA0436 and Heinz 1706 genomes.

Supplementary Note 2: Genome assembly of the 11 tomato accessions

We performed Pacific Biosciences (PacBio) single-molecule real-time (SMRT), high-throughput chromosome conformation capture (Hi-C) and Illumina sequencing for the 11 tomato accessions, generating a total of 400.5 Gb PacBio data, 457.2 Gb of Hi-C reads and 792.6 Gb of Illumina short-read sequences for these 11 accessions, representing an average of 40-, 48- and 80-fold genome coverage, respectively (**Supplementary Tables 1–3**). Based on *k*-mer analyses, we observed that the genome heterozygosity of *S. corneliomulleri* (0.98%), *S. peruvianum* (0.66%) and *S. chilense* (0.65%) were relatively high (> 0.5%), whereas the other wild tomato genomes were largely homozygous (**Supplementary Fig. 1**).

The initial assembly of PacBio long reads (~79 Gb, ~96-fold coverage) of *S. galapagense* 'LA0436' resulted in a contig N50 size of 15.5 Mb (**Table 1**). Scaffolding was subsequently performed using approximately 177 Gb (213-fold coverage) Bionano optical map sequences (**Supplementary Table 4**), leading to 195 scaffolds with an N50 length of 21.2 Mb (**Supplementary Table 5**). We then mapped Hi-C data to the assembled scaffolds to order and orient them into 12 chromosome-level super-scaffolds. After filling gaps using PacBio reads, the final assembly of LA0436 had a total length of 802 Mb and only 40 super-scaffolds (**Table 1** and **Supplementary Tables 4 and 5**). A higher degree of genome collinearity is retained between *S. galapagense* and 'Heinz 1706' than *S. pennellii*, in concordance with the previously reported evolutionary distance among these three species[10] (**Supplementary Fig. 2a,b**). The ten other tomato genomes had contig N50 sizes varying from 0.43 Mb (*S. chilense*) to 3.69 Mb (*S. pimpinellifolium*; **Table 1** and **Supplementary Tables 2, 6 and 7**).

Supplementary Note 3: Comparison between the tomato super-pangenome and the previously reported tomato pan-genome

In the super-pangenome, we observed that 75.0% (17,883 out of 23,839) of core genes identified in this study were shared in the previously reported tomato pan-genome[1], whereas the remaining 5,956 were uniquely regarded as core genes in this study. We also identified 1363, 238, 104 and 1274 genes that were uniquely present in clade I, II, III and IV, respectively. We found that genes specific to clade I display functions such as regulation of photoperiodism and malate transport, while some clade IV (red-fruited clade)-specific genes are involved in biological processes such as protein transport and localization (**Supplementary Fig. 5**).

Among the 9,320 non-redundant genes absent in the reported tomato pan-genome[1] (**Supplementary Table 14**), 5,158 arose due to non-collinear segments, and the remaining 4,162 were considered as additional alleles of existing genes. These genes displayed a similar distribution pattern among the *S. galapagense* genome, as compared with the whole-genome protein-coding genes (**Supplementary Fig. 8a–c**), and their coding length was significantly shorted than the total genes predicted in the 13 tomato genomes (911 bp versus 1,370 bp, $p < 2.2 \times 10^{-16}$, Kruskal Wallis test), with a lower exon number per gene (4 versus 5; **Supplementary Fig. 8d,e)**. We also observed that 69.3% (6,455 out of 9,320) of these genes exhibited low expression (transcripts per million [TPM] ≤ 0.5; **Supplementary Fig. 8f**). We further checked the distribution of these 9,320 genes in a tomato population of 321 accessions (226 *S. lycopersicum* [SLL], 85 *S. lycopersicum* var. *cerasiforme* [SLC] and 10 *S. pimpinellifolium* [SP] accessions)[6], and found that only a mean of 1,654 genes (17.7%) were found per accession, with 56.5% (5,265 out of 9,320) being uncaptured by any of the 321 tomatoes (**Supplementary Fig. 9**). These results suggest that the majority of the 9,320 genes are possibly unique to the distantly related wild tomato species. GO terms could be assigned to 3,374 out of the 9,320 genes (**Supplementary Table 14**), among which 122 were involved in defense response (**Supplementary Fig. 10**), suggesting potential values of further utilizing these genomic resources in tomato resistance breeding.

Supplementary Note 4: Features and examples of small genetic variants among wild and cultivated tomatoes

Most of the identified SNPs and InDels (81.7%) were localized in intergenic regions (0.063 variants per bp), whereas 3.3% resided within gene coding regions (0.039 variants per bp). Interestingly, we found that several functionally important genes carried clade-specific variants, including a photomorphogenesis-related gene *Sgal01g012480* (*PhyB1*) that encodes a red/far-red light photoreceptor, *Sgal01g016480* (*SlMYB12*) that regulates tomato fruit peel color, *Sgal02g028860* (*ZEP*), a gene encoding a zeaxanthin epoxidase and involved in abscisic acid biosynthesis, a fruit mass and plant architecture related gene *Sgal03g023990* (*SlKLUH*) that encodes a P450 enzyme CYP78A), and two carotenoid cleavage dioxygenase-encoding genes *CCD1*A (*Sgal01g019530*) and *CCD1B* (*Sgal01g019540*; **Supplementary Figs. 11–16**). These variants provide candidates for further investigation of mechanisms underlying possible clade-specific phenotypes in wild and cultivated tomatoes.

Supplementary Note 5: SVs that are only present in the tomato super-pangenome

To investigate the extent of diversity to which wild tomato species could contribute, we compared the identified SVs in this study with a previously reported tomato pan-SV map, which was built by long-read sequencing of 100 tomato accessions (89 SLL, SLC and SP accessions, and 11 *S. galapagense* and *S. cheesmaniae* accessions)[9]. In the pan-SV map, a total of 182,741 SVs were detected in the 89 cultivated and SP accessions, and the SV number increased to 226,215, when adding the 11 remaining wild tomato accessions (**Supplementary Fig. 23a**). In contrast, when incorporating the eight wild tomato species, the number of SVs identified in this study increased sharply from 38,054 to 224,447 (**Supplementary Fig. 23a**). Among the additional 186,393 SVs, 96.7% (180,314) were exclusively identified in this study. These SVs were mainly shorter than 5 kb (**Supplementary Fig. 23b,c**), of which 4,124 (2.3%) were localized within coding regions (CDS) of 3,515 genes. These genes were mainly enriched for biological processes such as defense response, response to stress and cell recognition (**Supplementary Table 21**). Our results suggest that the majority of SVs captured in this study was due to the inclusion of distantly related wild tomato species, revealing a rich amount of genetic diversity.

Supplementary Figures

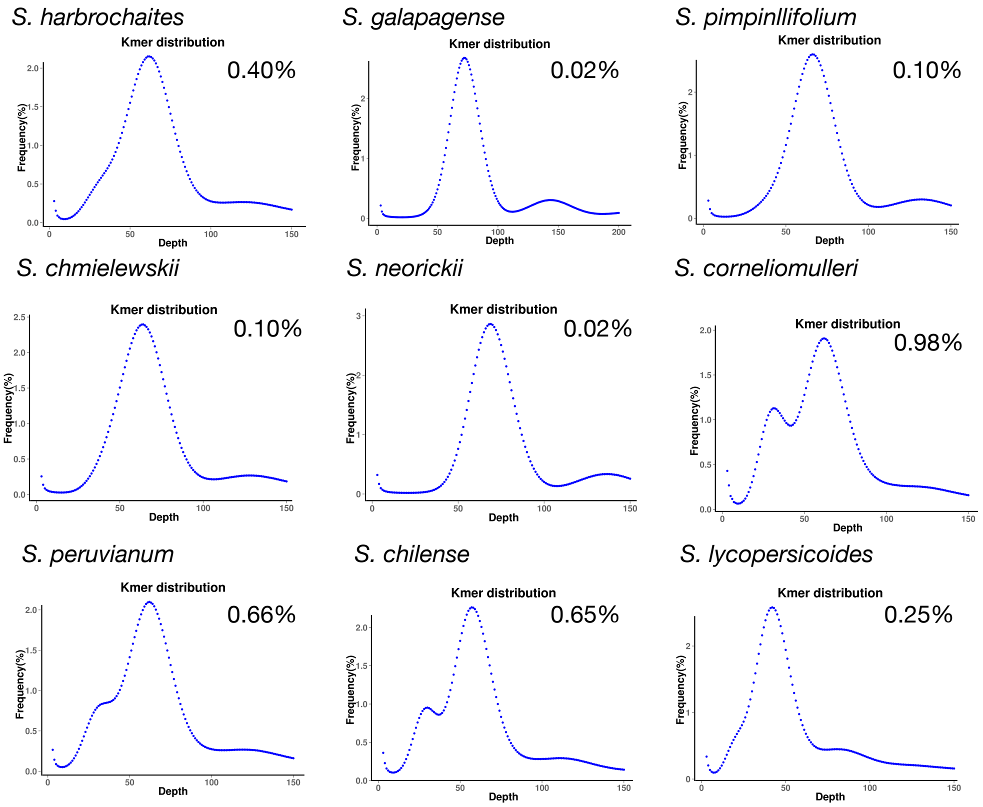

Supplementary Fig. 1. K-mer frequency distribution of genomes of the nine selected wild tomato species. Estimated heterozygosity rates are also marked.

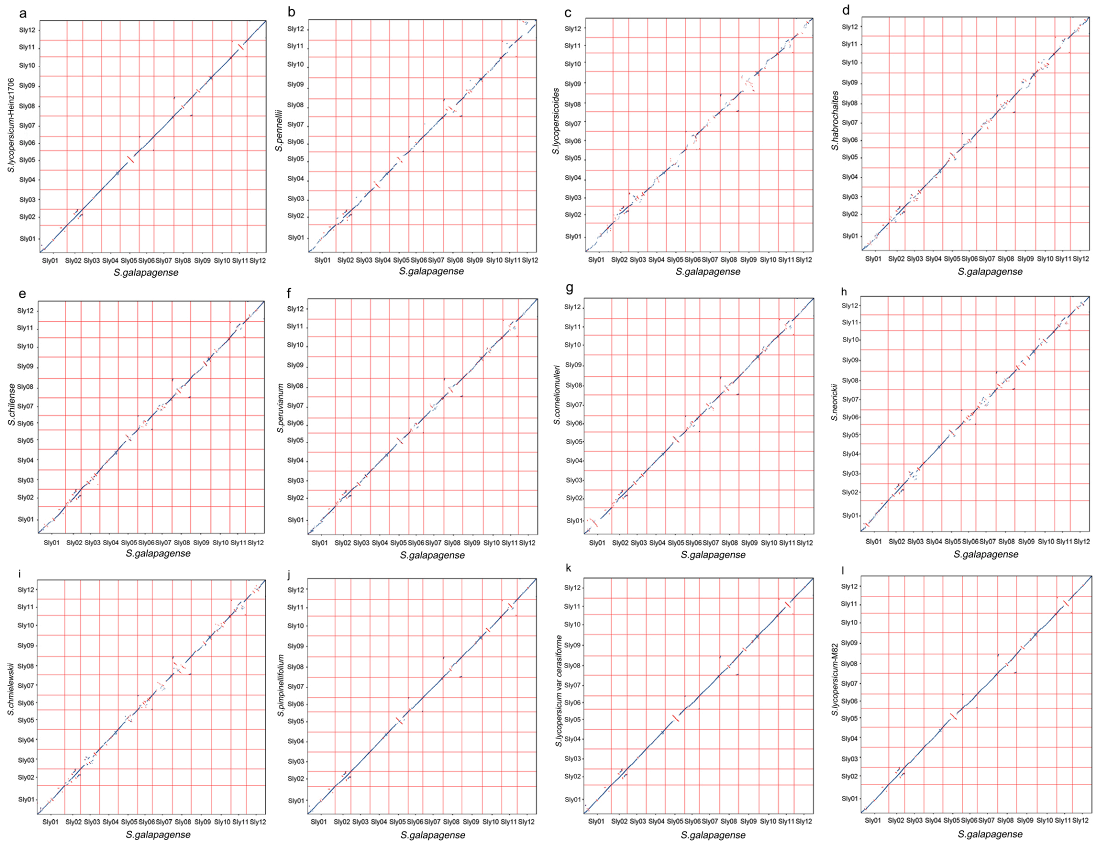

Supplementary Fig. 2. Genome alignment between *S. galapagense* (x-axis) and other 12 genomes in *Solanum* sect. *Lycopersicon* and *Solanum* sect. *Lycopersicoides* (y-axis). Alignments between the *S. galapagense* genome and the genome of *S. lycopersicum* cv. Heinz 1706 (a), *S. pennellii* (b), *S. lycopersicoides* (c), *S. habrochaites* (d), *S. chilense* (e), *S. peruvianum* (f), *S. corneliomulleri* (g), *S. neorickii* (h), *S. chmielewskii* (i), *S. pimpinellifolium* (j), *S. lycopersicum* var. *cerasiforme* (k) and *S. lycopersicum* cv. M82 (l) are illustrated in dot plots.

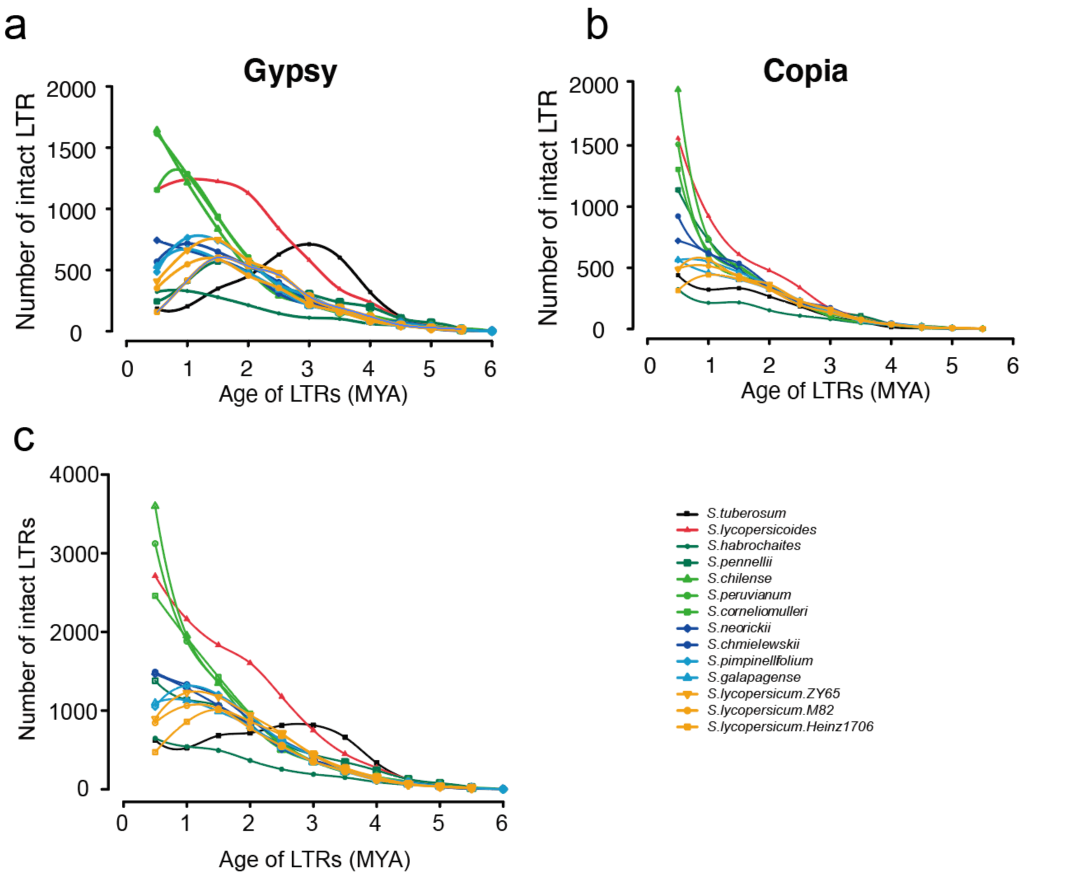

Supplementary Fig. 3. Distribution of insertion times for LTR retrotransposons in potato and different tomato genomes. a) Distribution of insertion times for *Gypsy* type LTRs, b) Distribution of insertion times for *Copia* type LTRs, c) Distribution of insertion times for *Gypsy* and *Copia*. Note that LTR-RTs of *S. lycopersicoides*, *S. chilense*, *S. peruvianum* and *S. corneliomulleri* might still be active now.

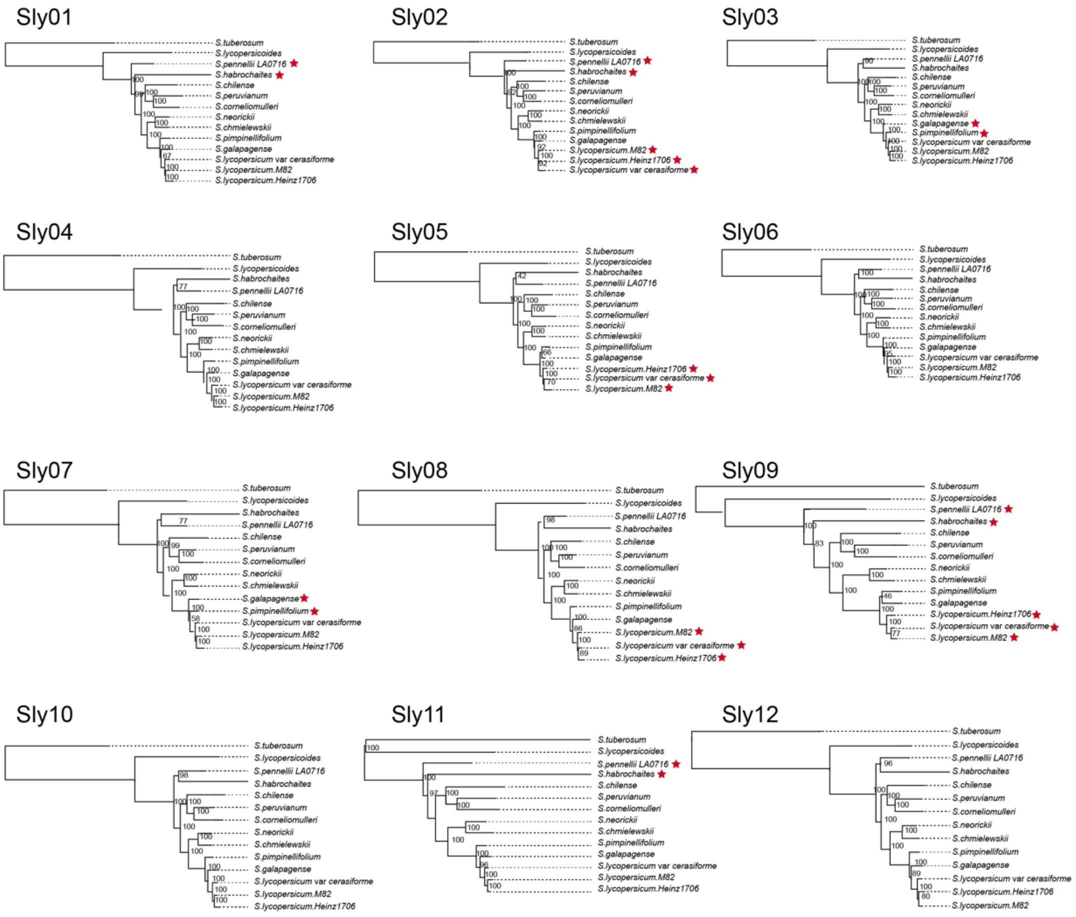

Supplementary Fig. 4. Maximum likelihood phylogenetic trees of each tomato chromosome. Species marked with red stars have conflict topology structure compared to the whole-genome phylogeny. All conflict topologies present in clade I and IV.

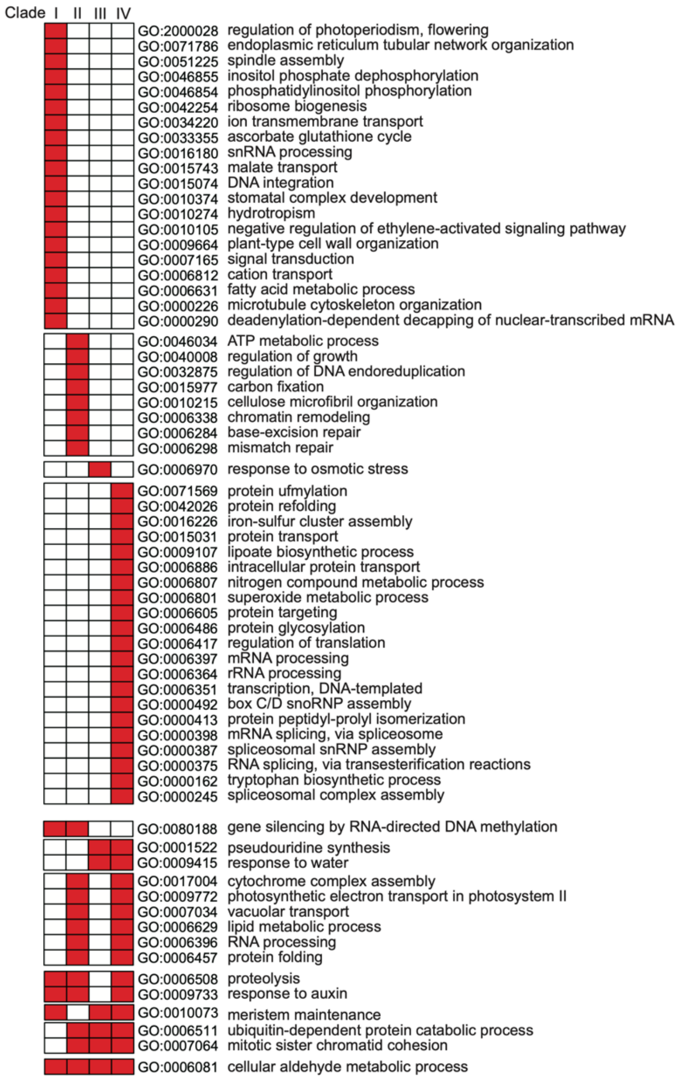

Supplementary Fig. 5. GO biological process terms predicted in the clade-specific genes.

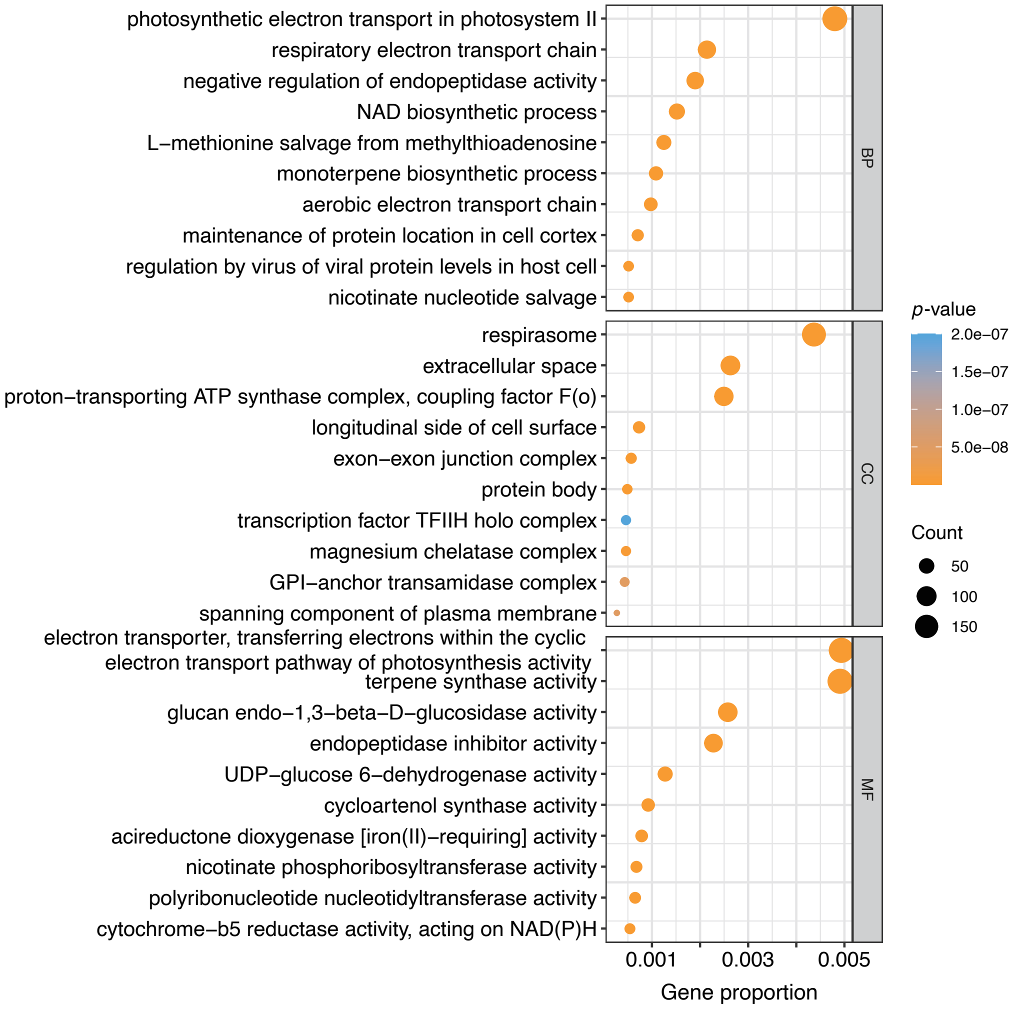

Supplementary Fig. 6. GO enrichment analysis of dispensable genes of the 13 tomato genomes. BP, biological process, CC, cellular component, MF, molecular function. The x-axis represents the proportion of genes falling into corresponding GO category among all the investigated genes. *P*-values were computed using two-tailed Fisher's exact test.

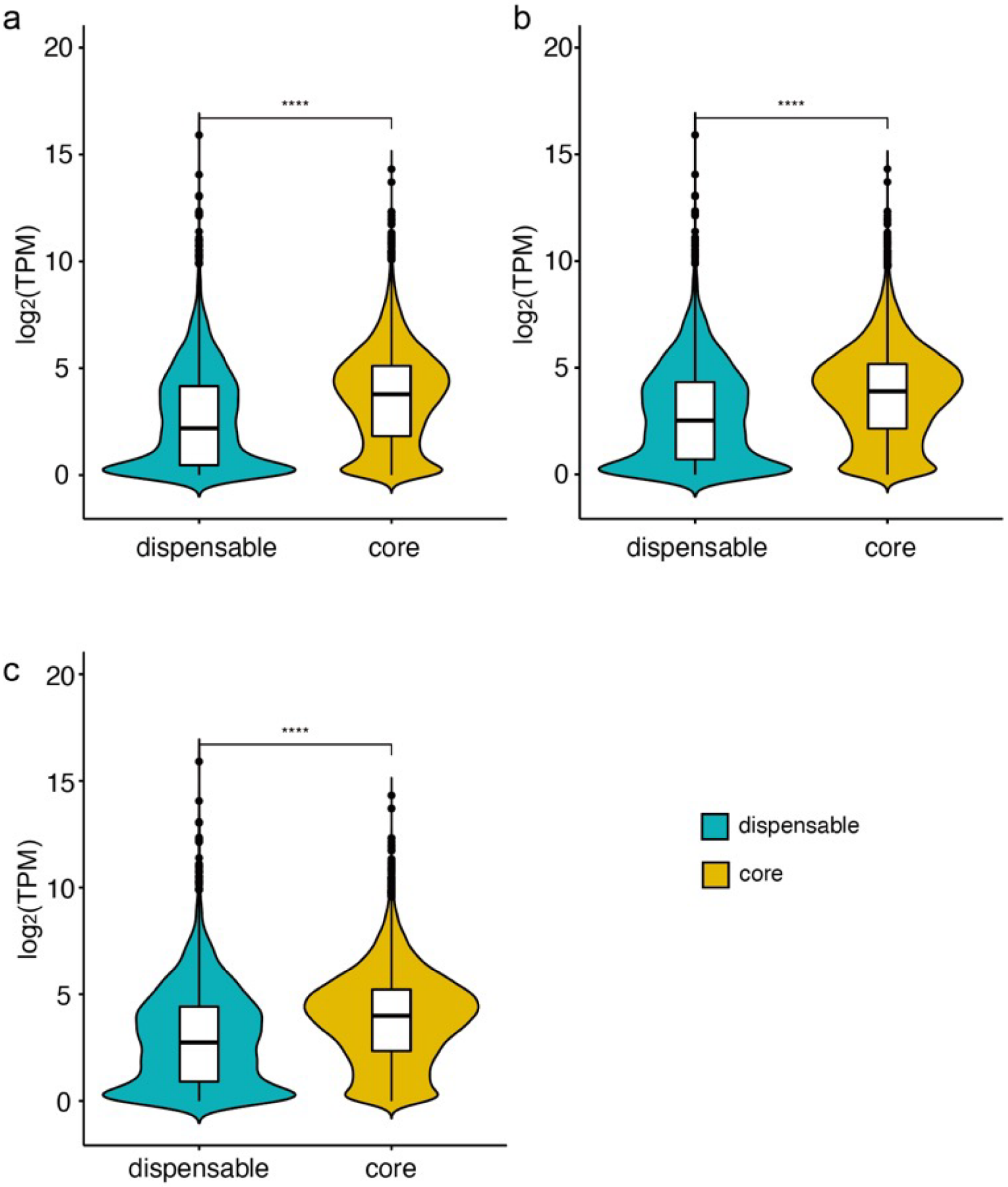

Supplementary Fig. 7. Expression levels of core and dispensable gene at different fruit ripening stages. a) mature green, b) breaker and c) red ripe stages. The 25% and 75% quartiles are shown as lower and upper edges of boxes, respectively, and central lines denote the median. Numbers of samples in core and dispensable genes are 19,403 and 13,370, respectively. The whiskers extend to 1.5 times the inter-quartile range. Data beyond the end of the whiskers are displayed as outlying dots. In **a**, **b** and **c**, p-values = 2.2×10^{-16}, which are computed using two-sided Wilcoxon rank-sum test. **** $p < 0.0001$. The density curves show the data distribution.

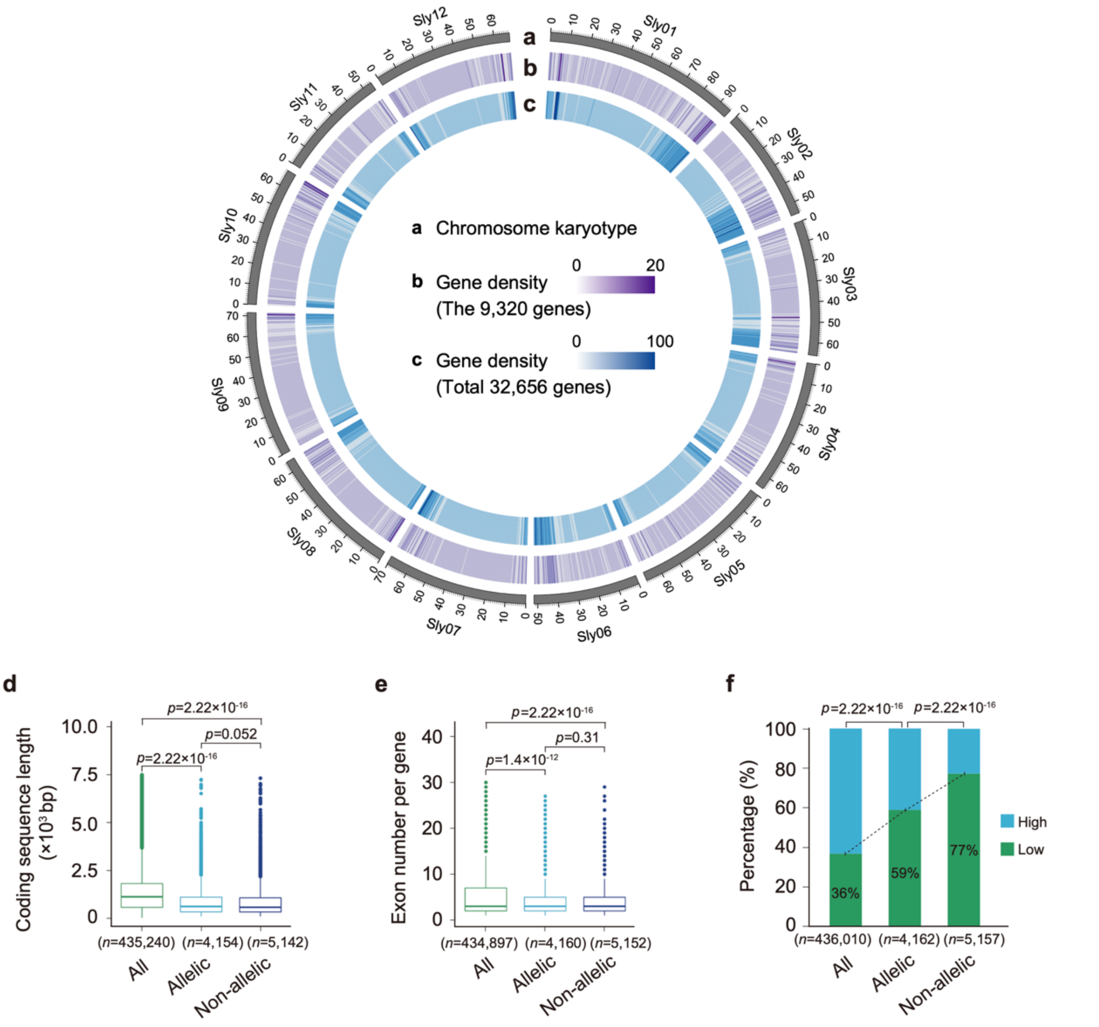

Supplementary Fig. 8. Features of the 9,320 genes identified in this study. a) Karyotype of the 12 tomato chromosomes using *S. galapagense* as the reference genome. b) Genome-wide distribution heatmap of the 9,320 genes identified in this study. c) Genome-wide distribution heatmap of the 32,656 genes predicted in the *S. galapagense* reference genome. d) Distribution of coding sequence (CDS) length of genes predicted in all the 13 tomato genomes, the 4,162 genes being additional alleles of existing genes (Allelic) and the 5,158 genes arose due to non-collinear segments (Non-allelic). Only genes with CDS ≤ 7.5 kb were considered. e) Number of exons per gene in genes predicted in all the 13 tomato genomes, allelic and non-allelic genes (only genes carrying exons fewer than 30 were considered). In **d** and **e**, the 25% and 75% quartiles are shown as lower and upper edges of boxes, respectively, and central lines denote the median. The whiskers extend to 1.5 times of the inter-quartile range. Data beyond the end of the whiskers are displayed as outliers. *P*-values were computed using the two-tailed Kruskal-Wallis test. f) Percentage of genes predicted in all the 13 tomato genomes, allelic and non-allelic genes showing high (transcripts per million (TPM) > 0.5) and low (TPM ≤ 0.5) levels of expression. *P*-values were computed using two-tailed Fisher's exact test.

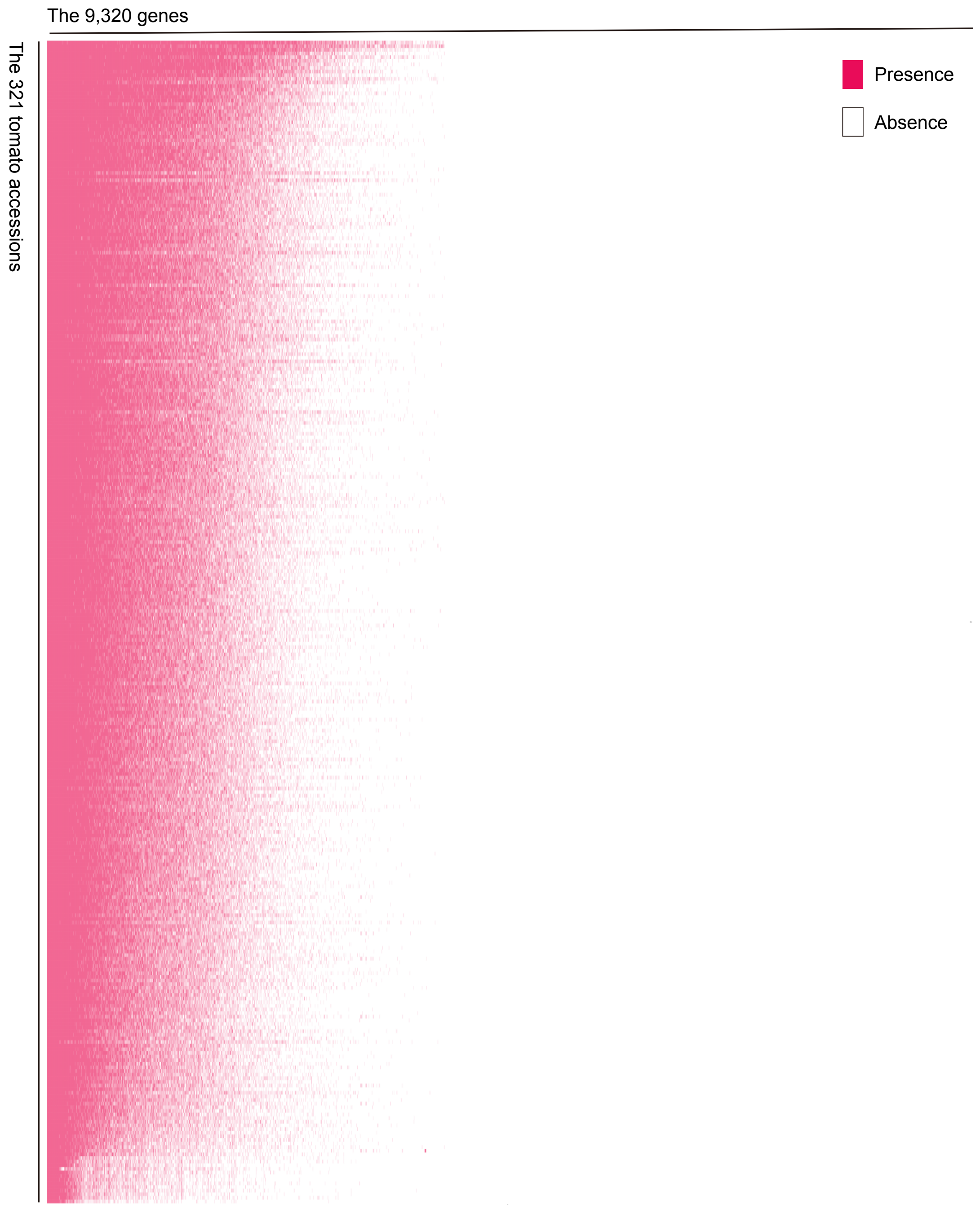

Supplementary Fig. 9. Distribution of the 9,320 non-redundant genes in a 321-line tomato population. Each row indicates one of the 321 tomato accessions and each column denotes one of the 9,320 genes.

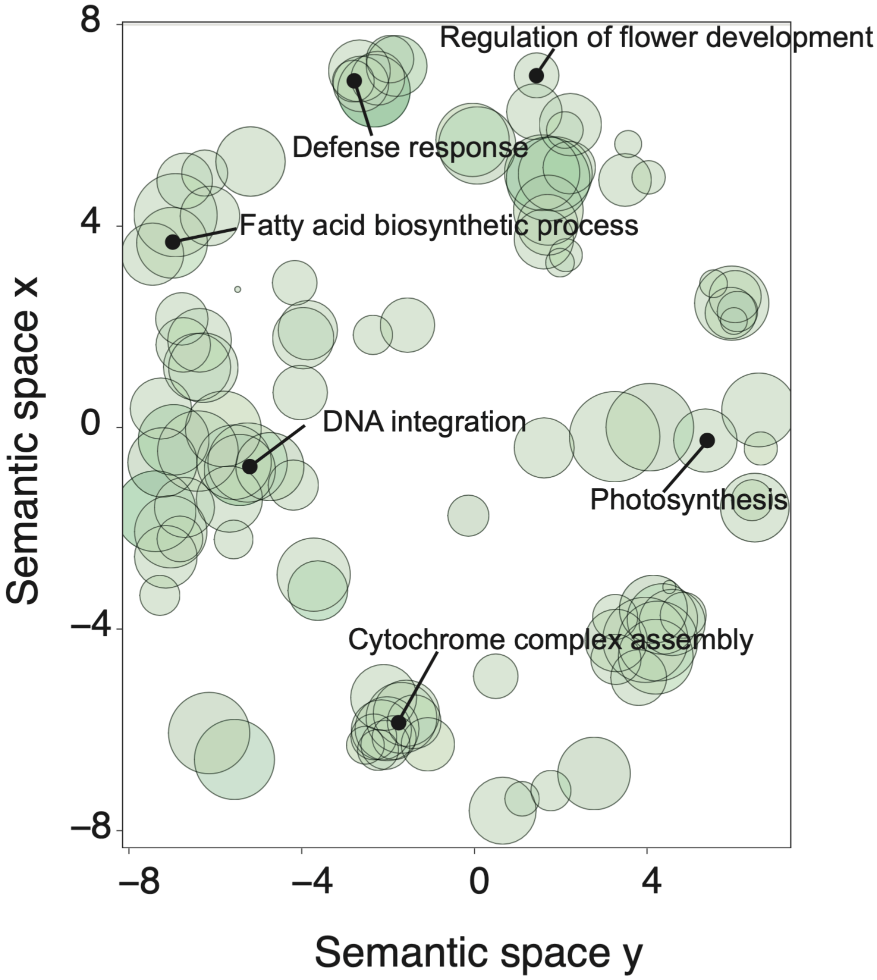

Supplementary Fig. 10. GO biological process terms of the 9,320 non-redundant genes only present in the pan-genome constructed in this study. Semantic representation of GO categories in terms of biological process of the 9,320 non-redundant genes only present in the pan-genome constructed in this study. Each bubble denotes one GO category and semantically similar GO terms were placed closer. The color of bubbles means the number of genes falling into the corresponding GO term (darker color denotes higher number of genes). Bubble size indicates the frequency of the GO category in the Gene Ontology Annotation (GOA) database[26]. The GO terms were clustered using REVIGO[27].

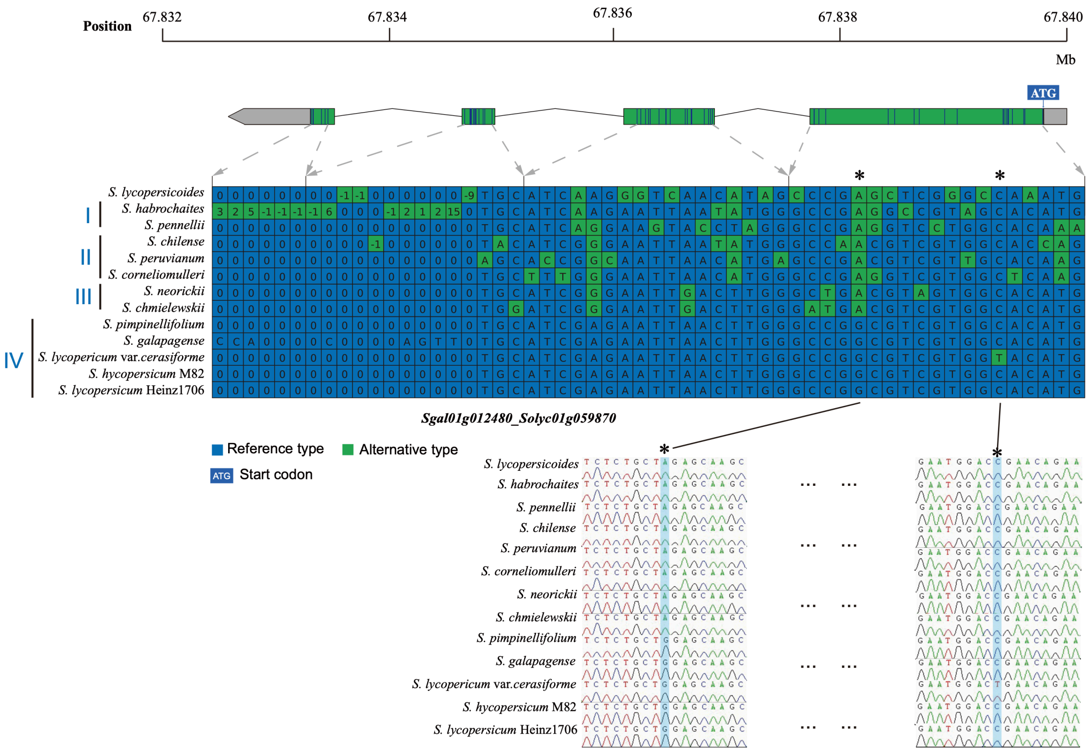

Supplementary Fig. 11. Haplotypes of *Sgal01g012480* among genomes of *S. lycopersicoides* and the 12 tomatoes. Clade IV species have the same haplotype except one C/T variation on the first exon of this gene in *S. lycopersicum* var. *cerasiforme*. The G/A allele at the first exon clearly separates clade IV species from other accessions. Sanger sequencing results for certain SNPs are also shown.

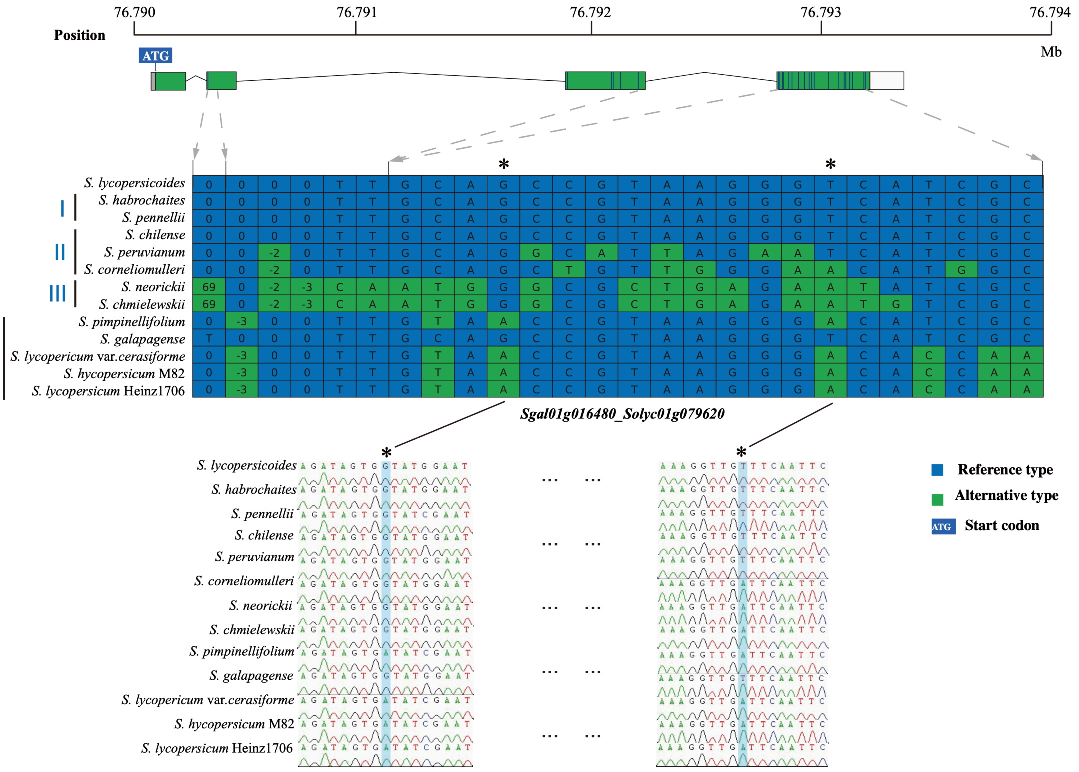

Supplementary Fig. 12. Haplotypes of *Sgal01g016480* among genomes of *S. lycopersicoides* and the 12 tomatoes. Except for *S. galapagenese*, other three genomes in clade IV have the same allele at four positions (sequenced two), which might be one of the evidences that cultivated tomatoes may be domesticated from *S. pimpinllifolium*. Sanger sequencing results for certain SNPs are also shown.

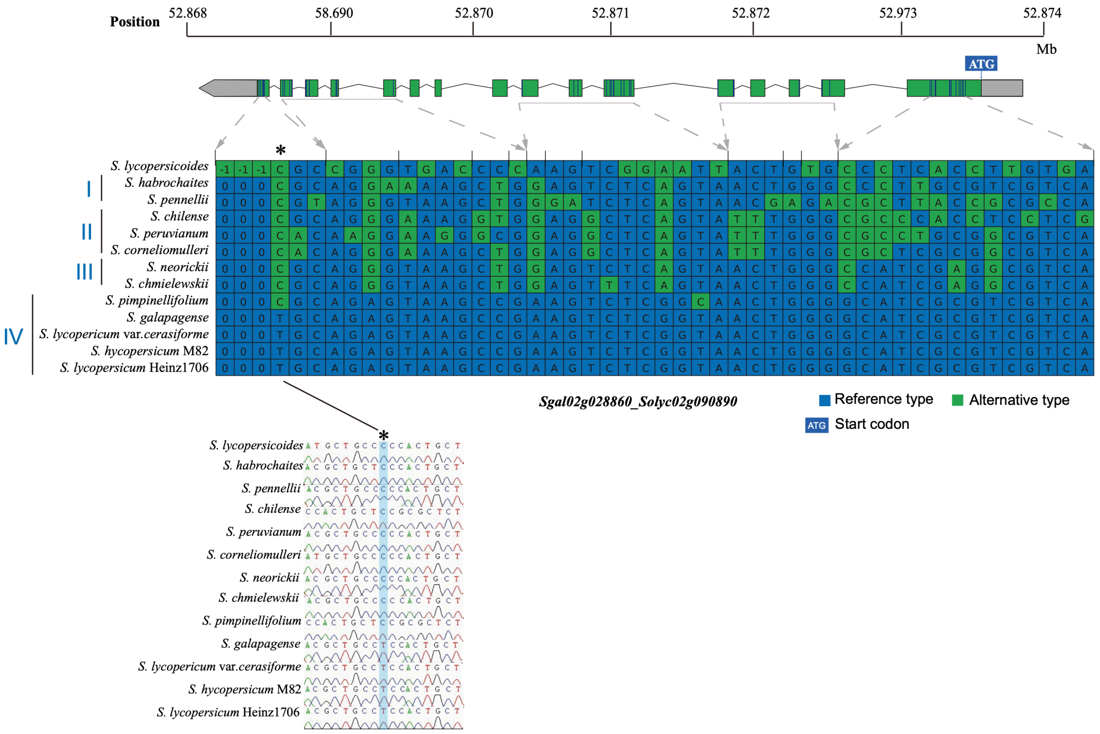

Supplementary Fig. 13. Haplotypes of *Sgal02g028860* among genomes of *S. lycopersicoides* and the 12 tomatoes. Except for *S. pimpinellifolium,* other species in clade IV have the same haplotype. Sanger sequencing results for the SNP are also shown.

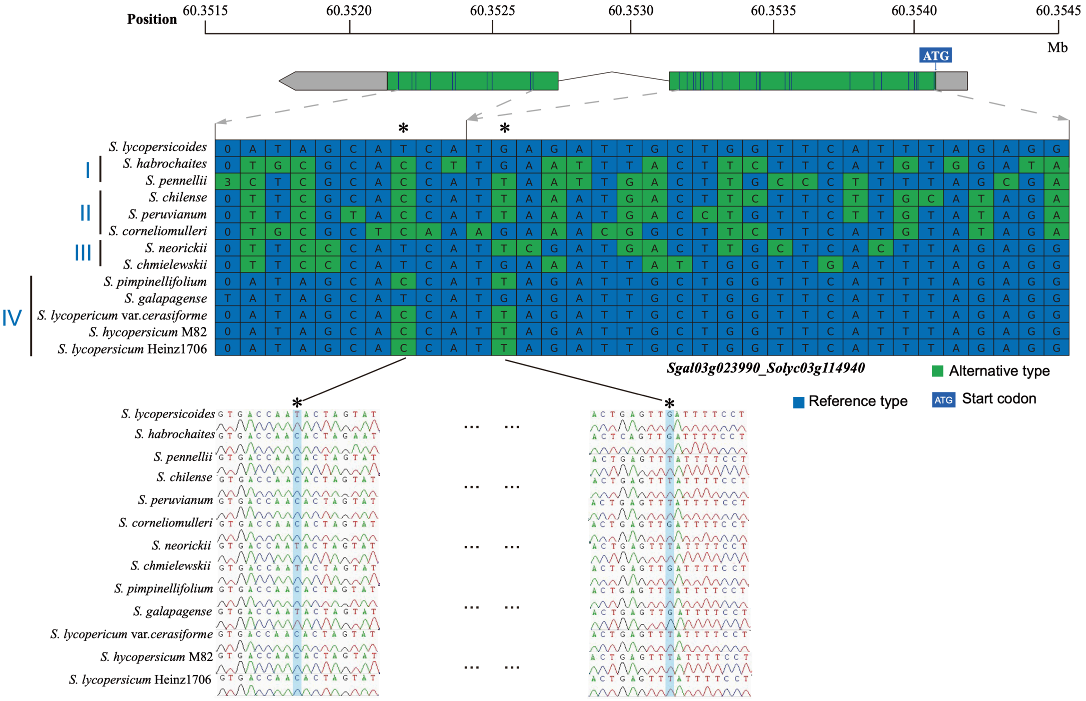

Supplementary Fig. 14. Haplotypes of *Sgal03g023990* among genomes of *S. lycopersicoides* and the 12 tomatoes. Two *S. galapagenese* specific allele are localized at the first and second exons. Sanger sequencing results for certain SNPs are also shown.

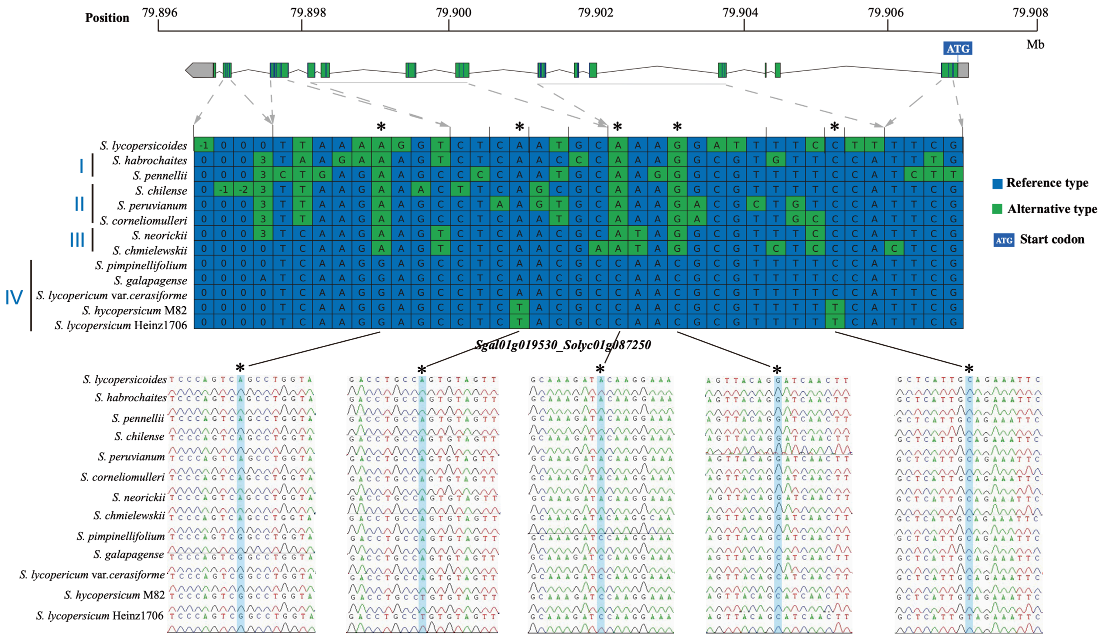

Supplementary Fig. 15. Haplotypes of *Sgal01g019530* among genomes of *S. lycopersicoides* and the 12 tomatoes. The C/G, C/A, G/A alleles clearly separate clade IV species from other wild relatives. Moreover, C/T and A/T alleles in the 5th and the 10th exons distinguish M82 and Heizn1706 to other species. Sanger sequencing results for certain SNPs are also shown.

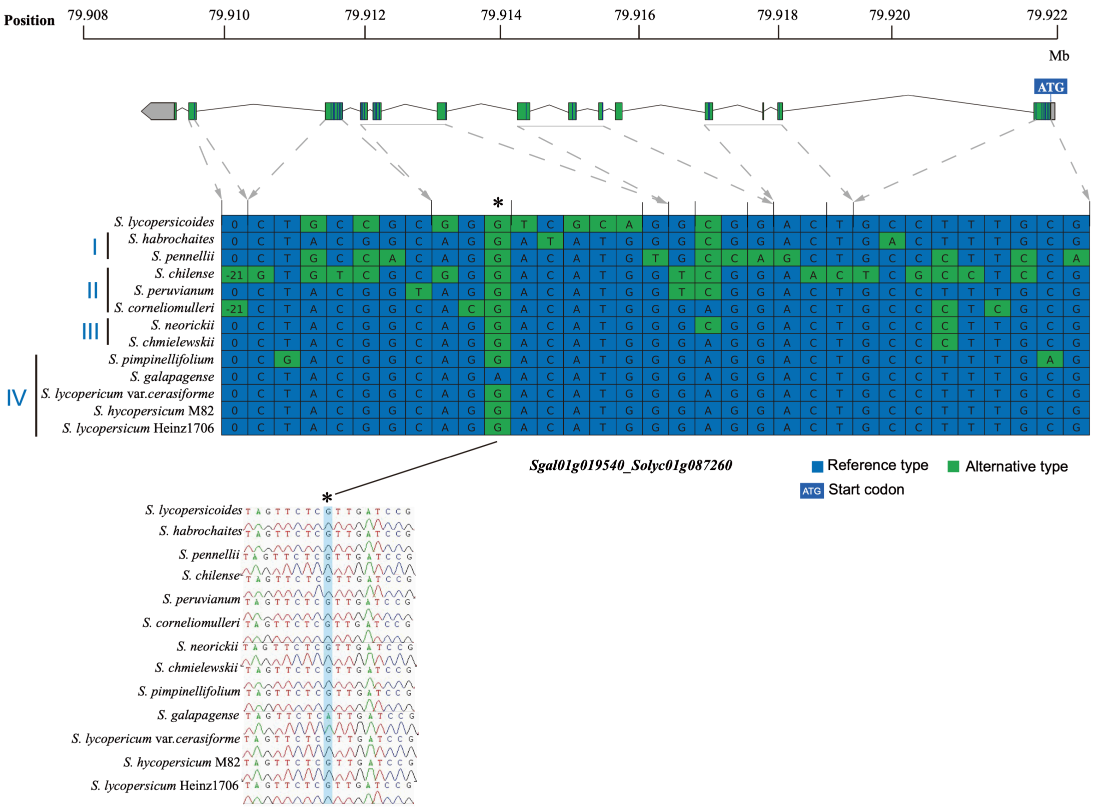

Supplementary Fig. 16. Haplotypes of *Sgal01g019540* among genomes of *S. lycopersicoides* and the 12 tomatoes. One *S. galapagenese*-specific allele exists at the 11th exon. Sanger sequencing results for the SNP are also shown.

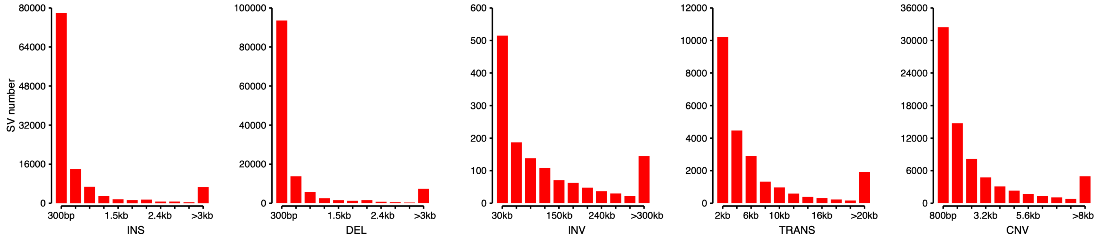

Supplementary Fig. 17. Length distribution of different types of structural variants.

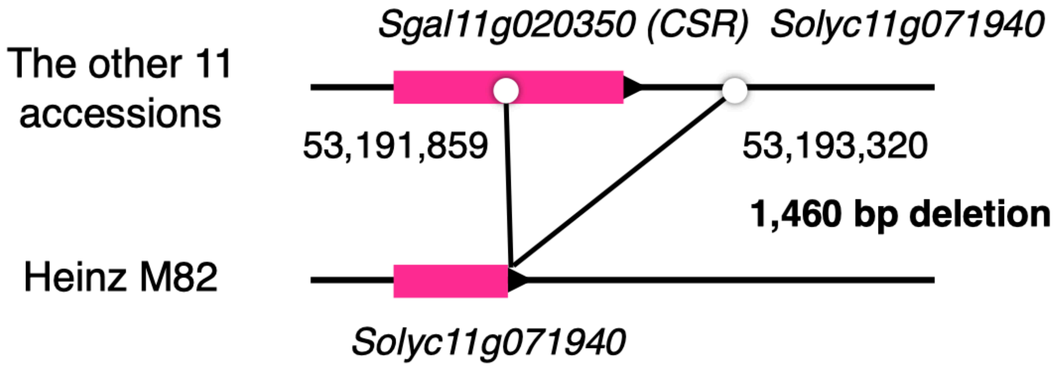

Supplementary Fig. 18. A 1,460-bp deletion within *CSR* gene among genomes of *S. lycopersicoides* and the 12 tomatoes. Two domesticated tomatoes (Heinz1706 and M82) have a 1,460-bp deletion at *CSR* gene body and its upstream regions.

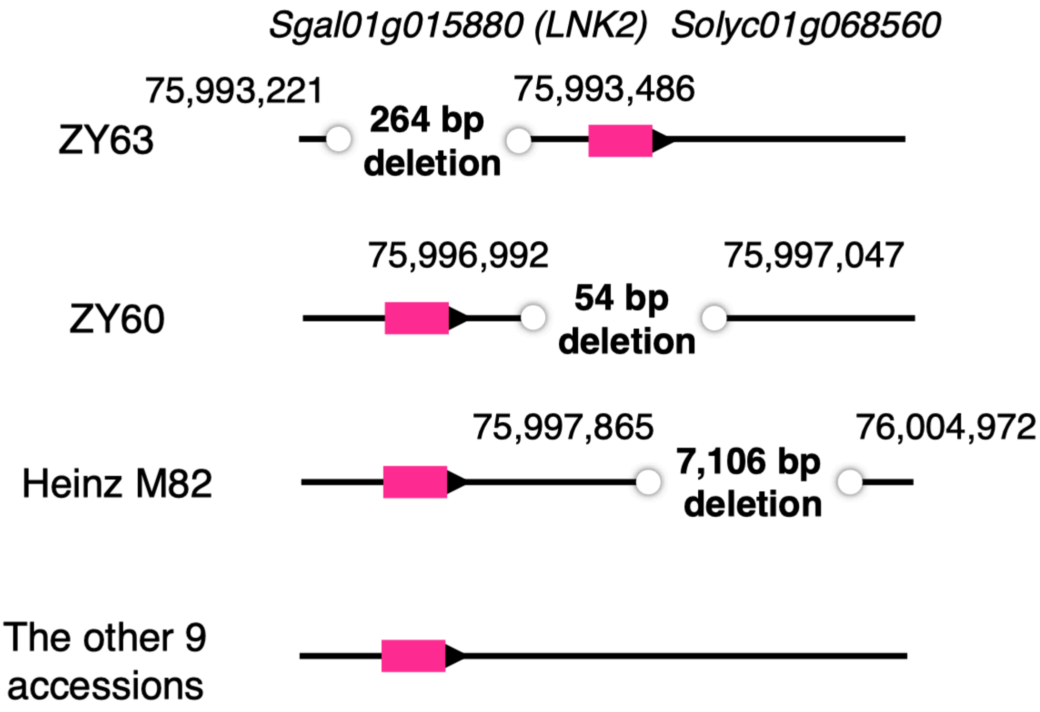

Supplementary Fig. 19. SVs within *LNK2* gene among genomes of *S. lycopersicoides* and the 12 tomatoes. Compared to *S. galapagenes*, a 7.1-kb deletion is detected at the upstream of the gene in Heinz1706 and M82. A 54-bp deletion is identified at the upstream of the gene in *S. chmieleswkii* (ZY60), and a 264-bp deletion occurs at the downstream of the gene in *S. chilense* (ZY63).

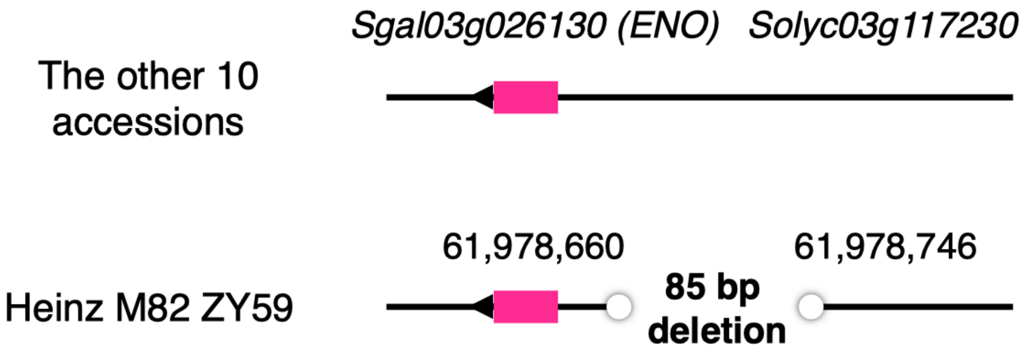

Supplementary Fig. 20. SVs within *ENO* gene among genomes of *S. lycopersicoides* and the 12 tomatoes. Two of domesticated tomatoes (Heinz1706 and M82) and a wild tomato (ZY59, *S. habrochaites*) have an 85-bp deletion at the downstream of *ENO*.

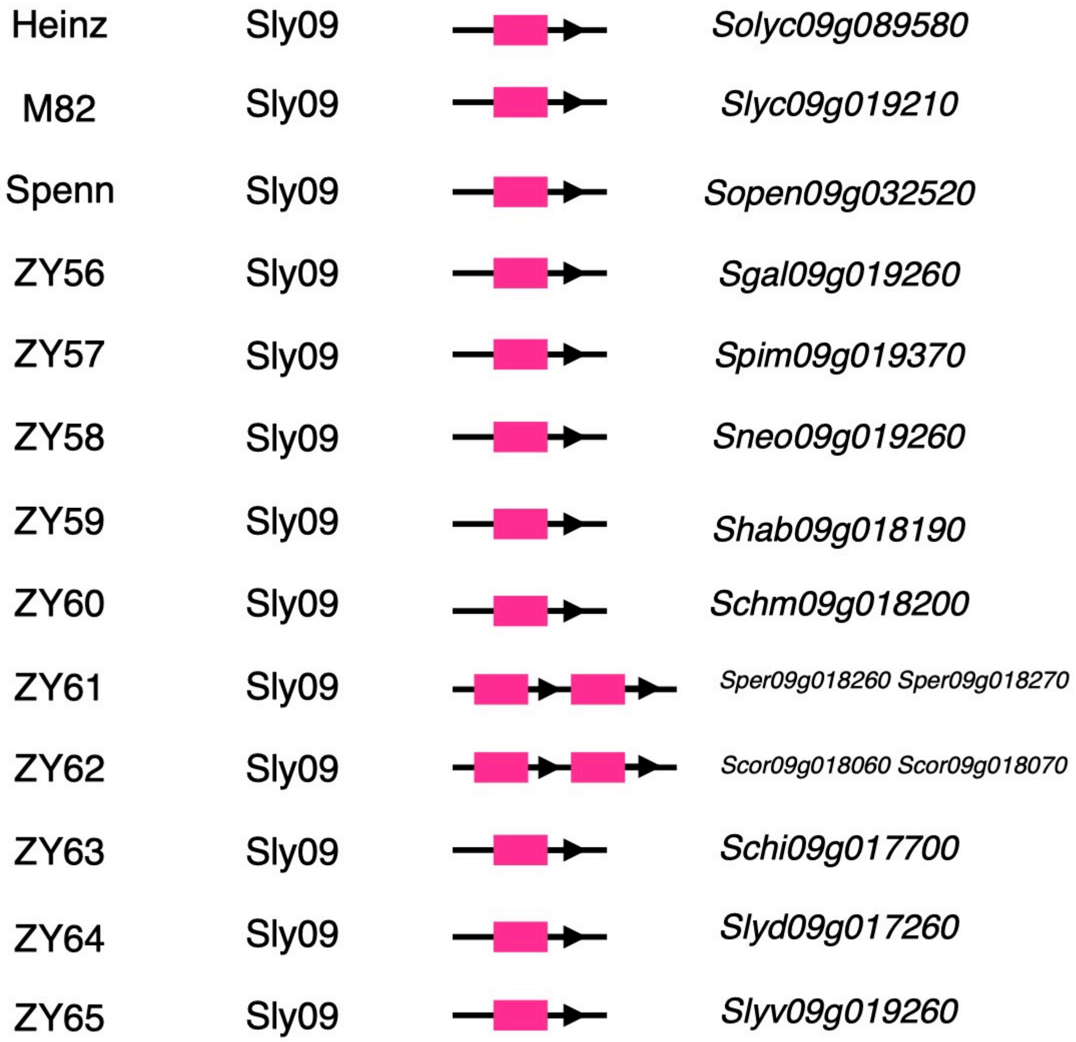

Supplementary Fig. 21. Copy number variation within *NSGT* gene among genomes of *S. lycopersicoides* and the 12 tomatoes. *S. peruvianum* (ZY61) and *S. corneliomulleri* (ZY62) have an additional copy of *NSGT* gene. M82, *S. lycopersicum*; Spenn, *S. pennellii*; ZY56, *S. galapagense*; ZY57, *S. pimpinellifolium*; ZY58, *S. neorickii*; ZY59, *S. habrochaites*; ZY60, *S. chmielewskii*; ZY61, *S. peruvianum*; ZY62, *S. corneliomulleri*; ZY63, *S. chilense*; ZY64, *S. lycopersicoides*; ZY65, *S. lycopersicum* var. *cerasiforme*.

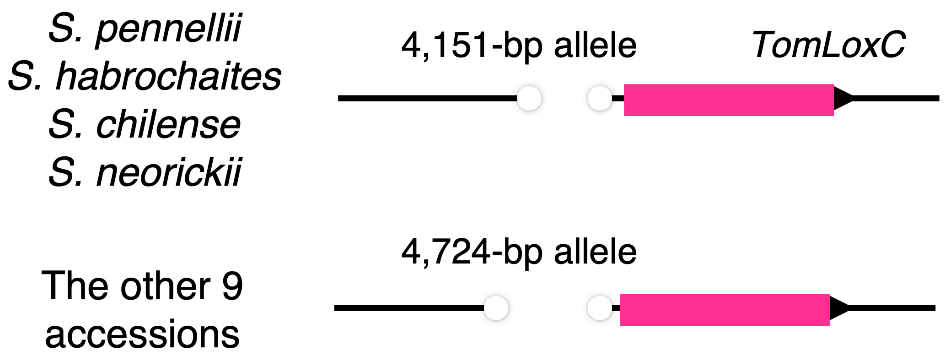

Supplementary Fig. 22. SVs within *TomLoxC* among genomes of *S. lycopersicoides* and the 12 tomatoes. The non-reference 4,151-bp allele upstream of *TomLoxC* is present in *S. pennellii*, *S. habrochaites*, *S. chilense* and *S. neorickii*, whereas the 4,724-bp allele is found in other tomato species.

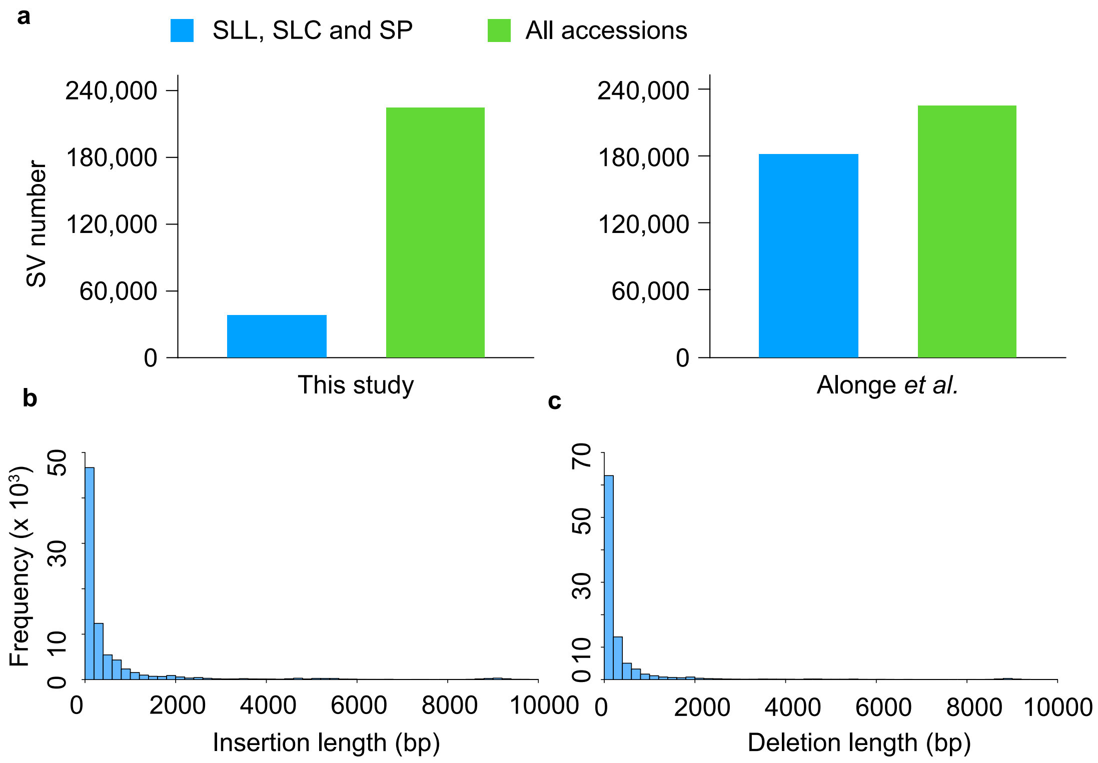

Supplementary Fig. 23. Comparison of SVs identified in this study and Alonge *et al.* a) Increase of SV number when including other wild accessions in this study and Along *et al*. SP, *S. pimpinellifolium* accessions, SLC, *S. lycopersicum* var. *cerasiforme* accessions, SLL, big-fruited *S. lycopersicum* accessions. b-c), Length distribution of insertions (**b**) and deletions (**c**) that could only be detected in this study due to the inclusion of more distantly related wild species.

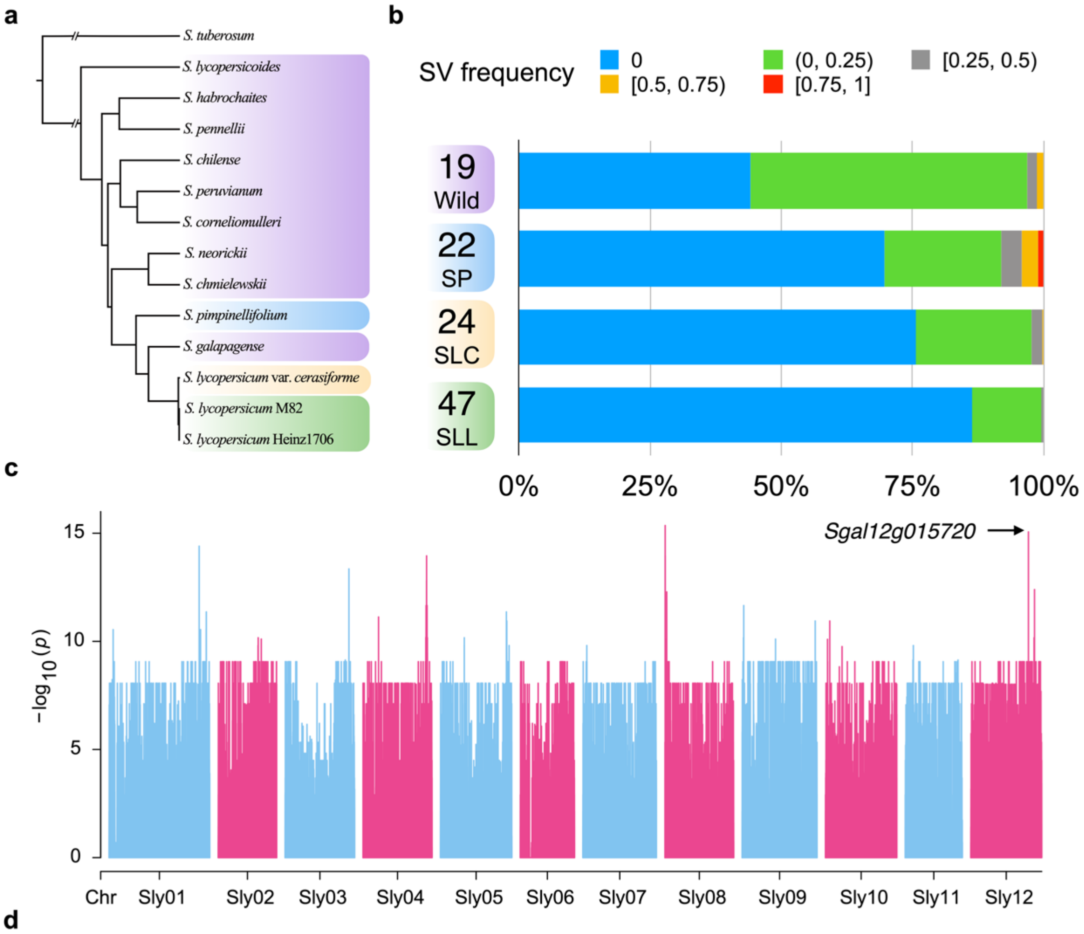

d

GO term	Description	Proportion	Proportion (background)	*P*-value
GO:0010073	meristem maintenance	17/787 (2.16%)	121/11222 (1.08%)	0.0045
GO:0048507	meristem development	17/787 (2.16%)	123/11222 (1.1%)	0.0053
GO:0015696	ammonium transport	3/787 (0.38%)	8/11222 (0.07%)	0.0147
GO:0072488	ammonium transmembrane transport	3/787 (0.38%)	8/11222 (0.07%)	0.0147
GO:0006357	regulation of transcription by RNA polymerase II	18/787 (2.29%)	153/11222 (1.36%)	0.021

Supplementary Fig. 24. Highly divergent SVs between wild and cultivated tomatoes a) Phylogeny of the ten wild and three cultivated tomato species used in this study, showing the separation of four different groups. b) SV presence frequency in Wild, SP, SLC and SLL groups. Wild, 19 distantly related wild tomato accessions from *S. galapagense*, *S. cheesmaniae*, *S. chmielewskii*, *S. neorickii*, *S. corneliomulleri*, *S. peruvianum*, *S. chilense*, *S. habrochaites*, *S. pennellii* and *S. lycopersicoides*, SP, 22 *S. pimpinellifolium* accessions, SLC, 24 *S. lycopersicum* var. *cerasiforme* accessions, SLL, 47 big-fruited *S. lycopersicum* accessions. c) *P*-values of two-tailed Fisher's exact test of SV presence frequencies between Wild and cultivated (SLC and SLL) groups across the 12 chromosomes. The gene showing the second highest -$\log_{10}$(*p*-values) (*Sgal12g015720*) is marked using a black arrow. d) Gene Ontology enrichment of the 2,585 genes impacted by the

8,094 highly divergent SVs between wild and cultivated tomatoes. In **c** and **d**, *P*-values were computed by two-tailed Fisher's exact test.

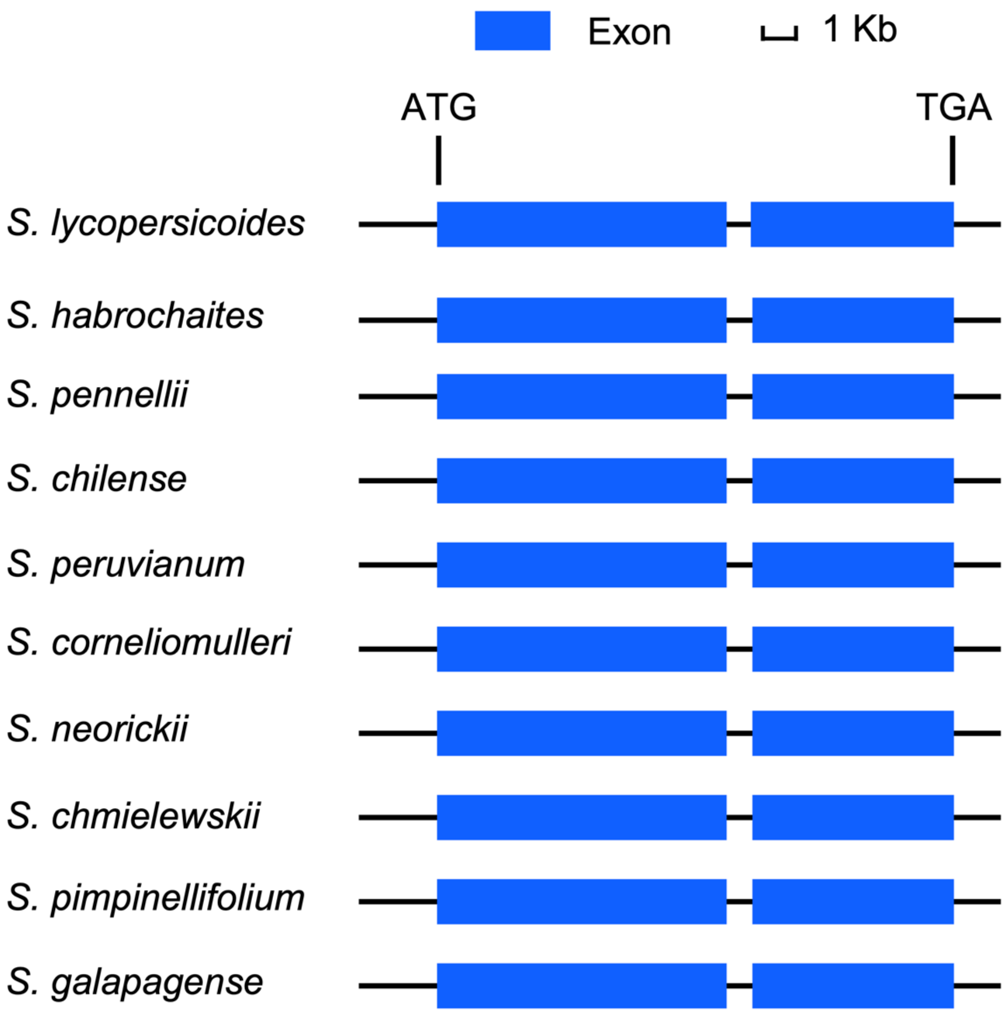

Supplementary Fig. 25. Structure of *Sgal12g015720* in the nine wild tomato species reported in this study.

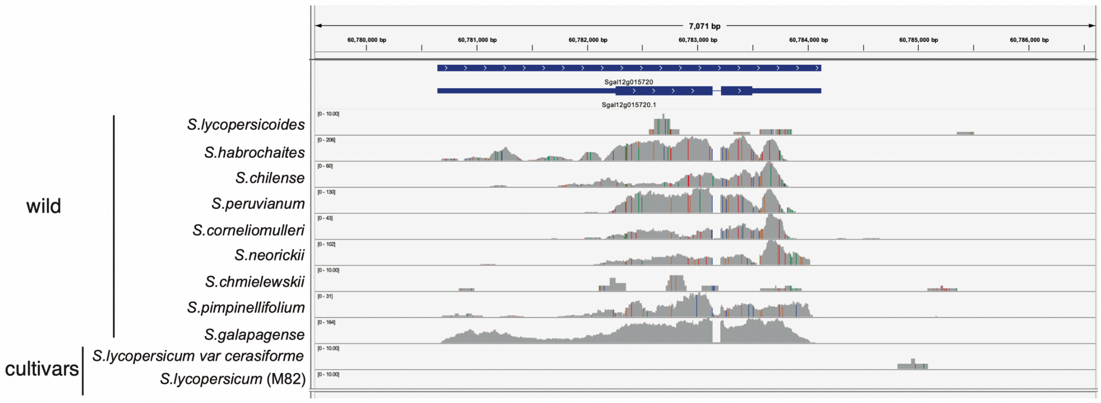

Supplementary Fig. 26. RNA-seq read mapping within the *Sgal12g015720* locus. The gene model of *Sgal12g015720* is shown in blue, in which exons are indicated by bold blue bars and the thin blue lines denote 5' and 3' UTRs. RNA-seq reads from the whole plants of nine wild tomato species and two cultivated tomato accessions are mapped against this region and genome coverages are shown in grey histograms.

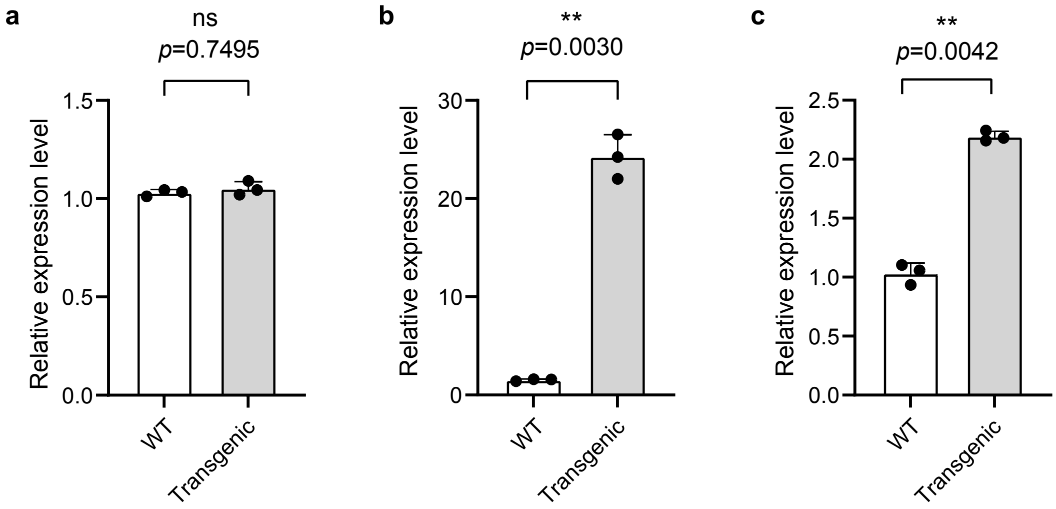

Supplementary Fig. 27. Quantification of relative expression of *Sgal12g015720* in roots, stems and leaves of wild-type and transgenic plants using quantitative real-time PCR (qRT-PCR). Relative expression levels are shown in roots (**a**), stems (**b**) and leaves (**c**). WT, the wild-type cultivated tomato "Micro Tom"; Transgenic, the overexpression transgenic lines of *Sgal12g015720*. Relative expression levels of *Sgal12g015720*in stems and leaves of the T_2 transformed lines are significantly higher than those in the wild-type (WT) plants. Data are shown in mean + SD, in which three biological replicates are conducted in each experiment. ns, no significant difference (p-value > 0.05); ** p-values < 0.01 in two-tailed Student's t-test.

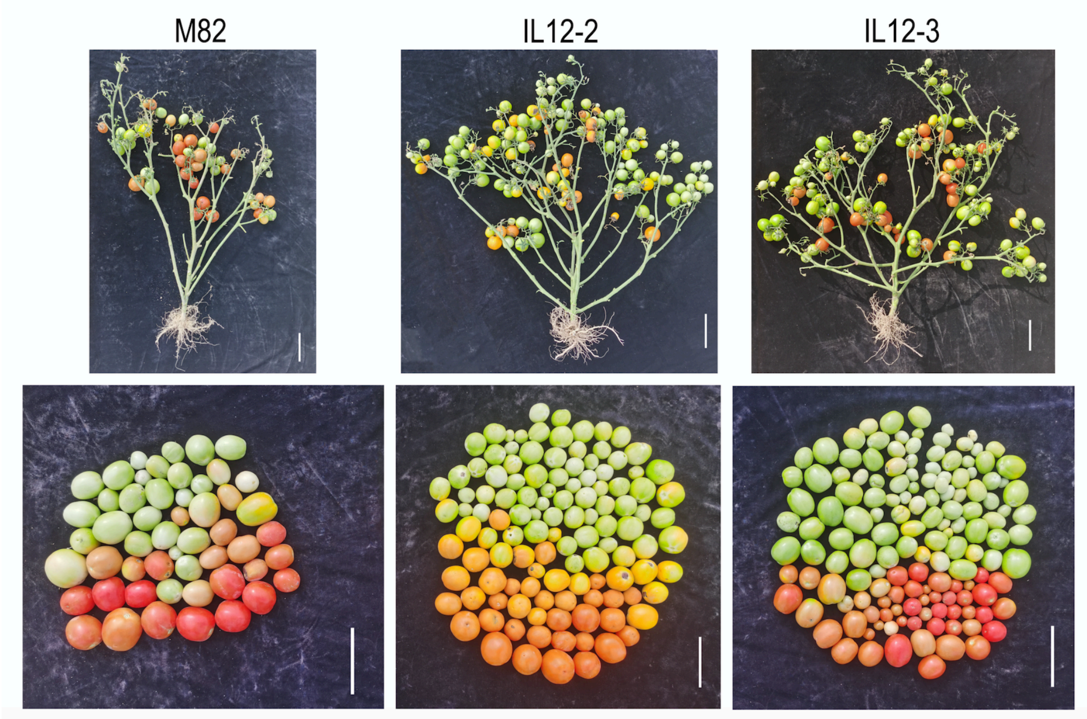

Supplementary Fig. 28. Plant architecture and fruits of M82 (the recurrent parent) and two introgressed lines (ILs). IL12-2 and IL12-3 are two ILs containing the *Sgal12g015720* segment. Leaves are removed to show fruit sets in the upper panel. Comparing to M82, the numbers of effective branches and total fruits increased markedly in these ILs. Scale bar = 10 cm.

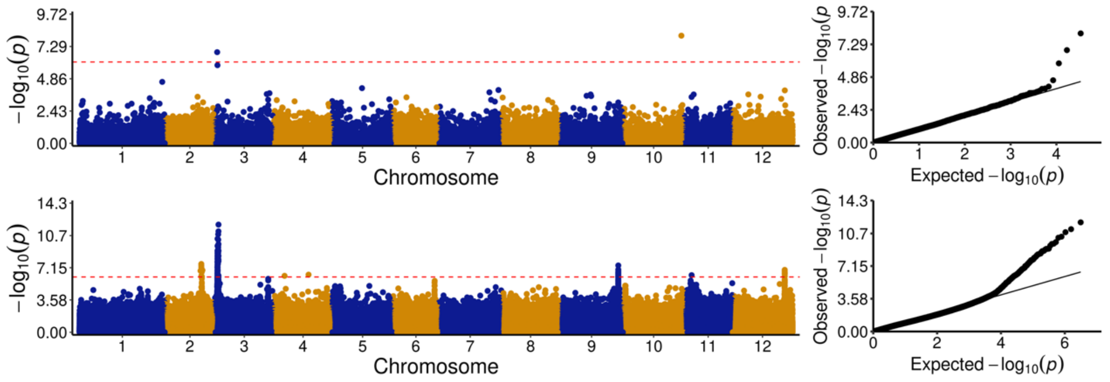

Supplementary Fig. 29. Genome-wide Manhattan plots and Quantile-Quantile plots for geranylacetone content in tomato fruits. Results of SV-based GWAS and SNP-based GWAS are shown in upper and lower panel, respectively. *P*-values are computed using efficient mixed-model association efficient mixed-model association (EMMAX) test. The expected *p* values in the Quantile-Quantile plots comply with a uniform distribution.

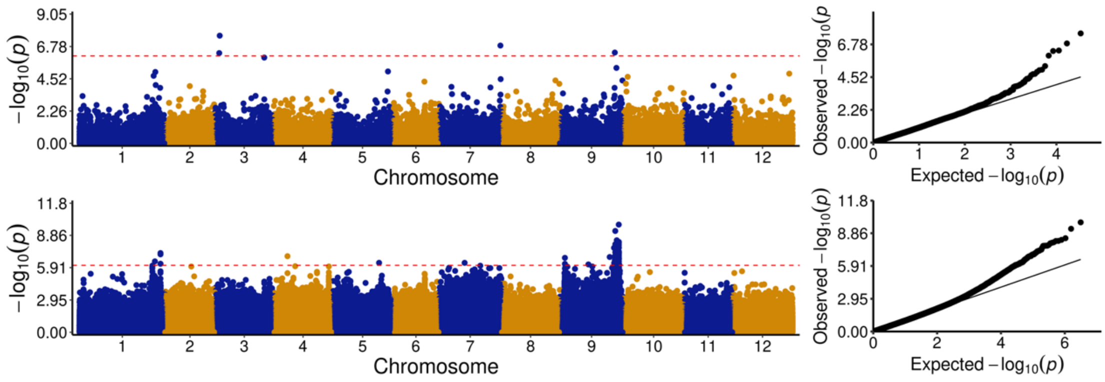

Supplementary Fig. 30. Genome-wide Manhattan plots and Quantile-Quantile plots for SlFM1955 (Kaempferol-sinapylglucosyl-xylosylrhamnoside) content in tomato fruits. Results of SV-based GWAS and SNP-based GWAS are shown in upper and lower panel, respectively. *P*-values are computed using efficient mixed-model association efficient mixed-model association (EMMAX) test. The expected *p* values in the Quantile-Quantile plots comply with a uniform distribution.

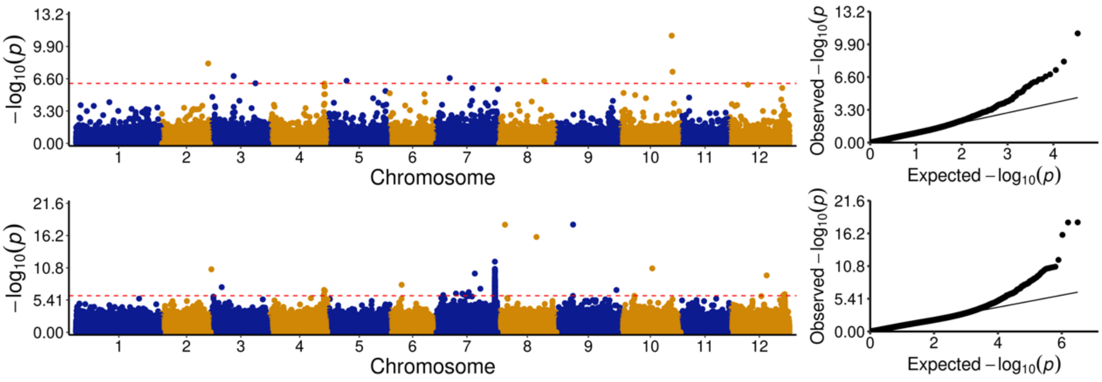

Supplementary Fig. 31. Genome-wide Manhattan plots and Quantile-Quantile plots for SlFM0306 (2'-deoxyadenosine monohydrate) content in tomato fruits. Results of SV-based GWAS and SNP-based GWAS are shown in upper and lower panel, respectively. *P*-values are computed using efficient mixed-model association efficient mixed-model association (EMMAX) test. The expected *p* values in the Quantile-Quantile plots comply with a uniform distribution.

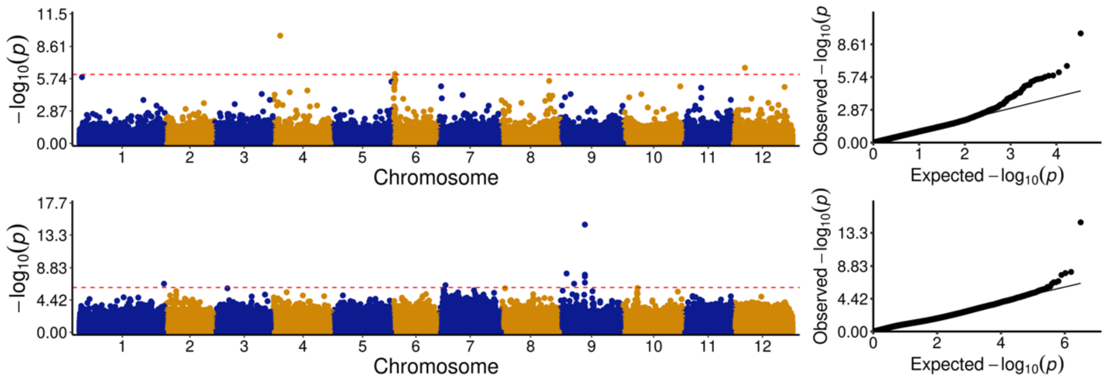

Supplementary Fig. 32. Genome-wide Manhattan plots and Quantile-Quantile plots for SlFM1209 (Tricin 7-*O*-hexoside) content in tomato fruits. Results of SV-based GWAS and SNP-based GWAS are shown in upper and lower panel, respectively. *P*-values are computed using efficient mixed-model association efficient mixed-model association (EMMAX) test. The expected *p* values in the Quantile-Quantile plots comply with a uniform distribution.

Supplementary References

1. Gao, L. *et al.* The tomato pan-genome uncovers new genes and a rare allele regulating fruit flavor. *Nat. Genet.* **51**, 1044–1051 (2019).
2. Camacho, C. *et al.* BLAST+: architecture and applications. *BMC bioinformatics* **10**, 1–9 (2009).
3. Ou, S. *et al.* Benchmarking transposable element annotation methods for creation of a streamlined, comprehensive pipeline. *Genome Biol.* **20**, 275 (2019).
4. Li, W. & Godzik, A. Cd-hit: a fast program for clustering and comparing large sets of protein or nucleotide sequences. *Bioinformatics* **22**, 1658–1659 (2006).
5. Chen, Y. *et al.* A Collinearity-Incorporating Homology Inference Strategy for Connecting Emerging Assemblies in the Triticeae Tribe as a Pilot Practice in the Plant Pangenomic Era. *Mol. Plant* **13**, 1694–1708 (2020).
6. Tieman, D. *et al.* A chemical genetic roadmap to improved tomato flavor. *Science* **355**, 391–394 (2017).
7. Li, H. & Durbin, R. Fast and accurate short read alignment with Burrows–Wheeler transform. *Bioinformatics* **25**, 1754–1760 (2009).
8. Li, H. *et al.* The sequence alignment/map format and SAMtools. *Bioinformatics* **25**, 2078–2079 (2009).
9. Alonge, M. *et al.* Major impacts of widespread structural variation on gene expression and crop improvement in tomato. *Cell* **182**, 145–161.e23 (2020).
10. Rodriguez, F., Wu, F., Ané, C., Tanksley, S. & Spooner, D. M. Do potatoes and tomatoes have a single evolutionary history, and what proportion of the genome supports this history? *BMC Evol. Biol.* **9**, 1–16 (2009).
