# Supplementary material for: Super-pangenome analyses highlight genomic diversity and structural variation across wild and cultivated tomato species
Source: Nat Genet. 2023 Apr 6;55(5):852–60. doi: 10.1038/s41588-023-01340-y (PMC10181942; doi:10.1038/s41588-023-01340-y)
Supplement: Supplementary file 2 — Reporting Summary [file 41588_2023_1340_MOESM2_ESM.pdf]

nature research

Corresponding author(s): Qinghui Yu, Hongbo Li and Huan Wang

Last updated by author(s): Dec 6, 2022

Reporting Summary

Nature Research wishes to improve the reproducibility of the work that we publish. This form provides structure for consistency and transparency in reporting. For further information on Nature Research policies, see our Editorial Policies and the Editorial Policy Checklist.

Statistics

For all statistical analyses, confirm that the following items are present in the figure legend, table legend, main text, or Methods section.

n/a	Confirmed	
☐	☒	The exact sample size (*n*) for each experimental group/condition, given as a discrete number and unit of measurement
☐	☒	A statement on whether measurements were taken from distinct samples or whether the same sample was measured repeatedly
☐	☒	The statistical test(s) used AND whether they are one- or two-sided<br>*Only common tests should be described solely by name; describe more complex techniques in the Methods section.*
☒	☐	A description of all covariates tested
☒	☐	A description of any assumptions or corrections, such as tests of normality and adjustment for multiple comparisons
☐	☒	A full description of the statistical parameters including central tendency (e.g. means) or other basic estimates (e.g. regression coefficient) AND variation (e.g. standard deviation) or associated estimates of uncertainty (e.g. confidence intervals)
☐	☒	For null hypothesis testing, the test statistic (e.g. *F*, *t*, *r*) with confidence intervals, effect sizes, degrees of freedom and *P* value noted<br>*Give P values as exact values whenever suitable.*
☒	☐	For Bayesian analysis, information on the choice of priors and Markov chain Monte Carlo settings
☒	☐	For hierarchical and complex designs, identification of the appropriate level for tests and full reporting of outcomes
☒	☐	Estimates of effect sizes (e.g. Cohen's *d*, Pearson's *r*), indicating how they were calculated

Our web collection on statistics for biologists contains articles on many of the points above.

Software and code

Policy information about availability of computer code

Data collection	No software was used to collect data. Data were sequenced from PacBio Sequel and Illumina NovaSeq 6000.
Data analysis	We used publicly available and appropriately cited software as described. No commercial software or code was used in this study. Software are listed as follows: Canu (v1.5), Pilon (version 1.22), IrysSolve (v3.5_12162019, https://bionanogenomics.com/support/software-downloads/), PBJelly (version 15.8.24), BWA (version 0.7.10-r789), HiC-Pro (v2.8.1), LACHESIS (v1.0), BUSCO (version 5.2.0), Trinity (v2.8.5), BLAST (v 2.12.0+), RepeatScout (version 1.0.5), LTR-FINDER (version 1.05), MITE-hunter (version 1.0), PILER-DF (version 1.0), REPET (version 2.5), RepeatMasker (version 4.0.5), GeMoMa (version 1.3.1), GlimmerHMM (version 3.0.4), HISAT2 (version 2.0.4), Stringtie (version 1.2.3), BLAT (v.36), PASA (version 2.0.4), TopHat (version 2.0.12), Cufflinks (version 2.2.1), Transdecoder (version 2.0), EVidenceModeler (version 1.1.1), Blast2GO (version 4.1.8), GMAP (version 2015-06-12), OrthoFinder (v2.5.2), EDTA (v1.9.4), Cd-hit (v4.8.1), quota-alignment (version 1.0), MUSCLE (version 3.8.31), phyML (version v3.3.20190909), PAML (version 4.7b), MUMmer (version 4.0.0beta2), SVMU (v0.4-alpha), SyRI (v1.2), minimap2 (v2.21-r1071), SURVIVOR (v1.0.6), R (v4.03), vg (v1.38.0), GATK (v4.1.4.1), PLINK (v1.9.0b4.6), EMMAX (v20120210), Genetic type 1 Error Calculator (v0.2), EMBOSS package (v6.6.0).<br>Custom codes and scripts are available at https://github.com/HongboDoll/TomatoSuperPanGenome and https://doi.org/10.5281/zenodo.7396707.

For manuscripts utilizing custom algorithms or software that are central to the research but not yet described in published literature, software must be made available to editors and reviewers. We strongly encourage code deposition in a community repository (e.g. GitHub). See the Nature Research guidelines for submitting code & software for further information.

Data

Policy information about availability of data

All manuscripts must include a data availability statement. This statement should provide the following information, where applicable:

- Accession codes, unique identifiers, or web links for publicly available datasets
- A list of figures that have associated raw data
- A description of any restrictions on data availability

All assembled genome sequences and their annotation are publicly accessible through our database (http://caastomato.biocloud.net). We have also deposited the genome assemblies in the NCBI GenBank under the accession number PRJNA809001. Raw PacBio data, transcriptome and Hi-C sequencing reads have been deposited into NCBI sequence read archive (SRA) (https://www.ncbi.nlm.nih.gov/sra/) under BioProject accession number PRJNA756391. Whole-genome sequencing data were downloaded from NCBI (BioProjects: PRJNA259308, PRJNA353161, PRJNA454805 and PRJEB5235). The RepBase database was downloaded from https://www.girinst.org/server/RepBase/index.php.

Field-specific reporting

Please select the one below that is the best fit for your research. If you are not sure, read the appropriate sections before making your selection.

☒ Life sciences ☐ Behavioural & social sciences ☐ Ecological, evolutionary & environmental sciences

For a reference copy of the document with all sections, see nature.com/documents/nr-reporting-summary-flat.pdf

Life sciences study design

All studies must disclose on these points even when the disclosure is negative.

Sample size	We selected 11 tomato accessions, representing nine wild and one cultivated tomato species. The logic of this selection was based on the extant wild (12) and cultivated (1) tomato species that are collectible.
Data exclusions	No samples were excluded in this study. Filters applied to eliminate low-quality sequencing data and genetic variants were properly described in the Methods section.
Replication	Three biological replicates with two technical replicates were used in the qRT-PCR experiment. Three independent T2 transgenic lines were generated for the estimation of single fruit weight, transverse diameter, longitudinal diameter, total fruit number and total fruit weight, in which three independent wild-type plants were also measured. All replications were successful and were used.
Randomization	For each tomato individual, the sampling process for genome DNA/RNA sequencing was randomly conducted. All WT and transgenic plants were exposed to the same growth condition and treatment.
Blinding	Blinding is not necessary for genome sequencing and assembly, since the investigators know which tomato species they were handling. The investigators were blinded to group allocation during collecting data from WT and transgenic tomato plants.

Reporting for specific materials, systems and methods

We require information from authors about some types of materials, experimental systems and methods used in many studies. Here, indicate whether each material, system or method listed is relevant to your study. If you are not sure if a list item applies to your research, read the appropriate section before selecting a response.

Materials & experimental systems

n/a	Involved in the study
☒	☐ Antibodies
☒	☐ Eukaryotic cell lines
☒	☐ Palaeontology and archaeology
☒	☐ Animals and other organisms
☒	☐ Human research participants
☒	☐ Clinical data
☒	☐ Dual use research of concern

Methods

n/a	Involved in the study
☒	☐ ChIP-seq
☒	☐ Flow cytometry
☒	☐ MRI-based neuroimaging
