# Supplementary material for: Super-pangenome analyses highlight genomic diversity and structural variation across wild and cultivated tomato species
Source: Nat Genet. 2023 Apr 6;55(5):852–60. doi: 10.1038/s41588-023-01340-y (PMC10181942; doi:10.1038/s41588-023-01340-y)

Unprocessed gels for Fig. 3b.
PCR validation of the 244-bp deletion in ten wild and three domesticated tomatoes.

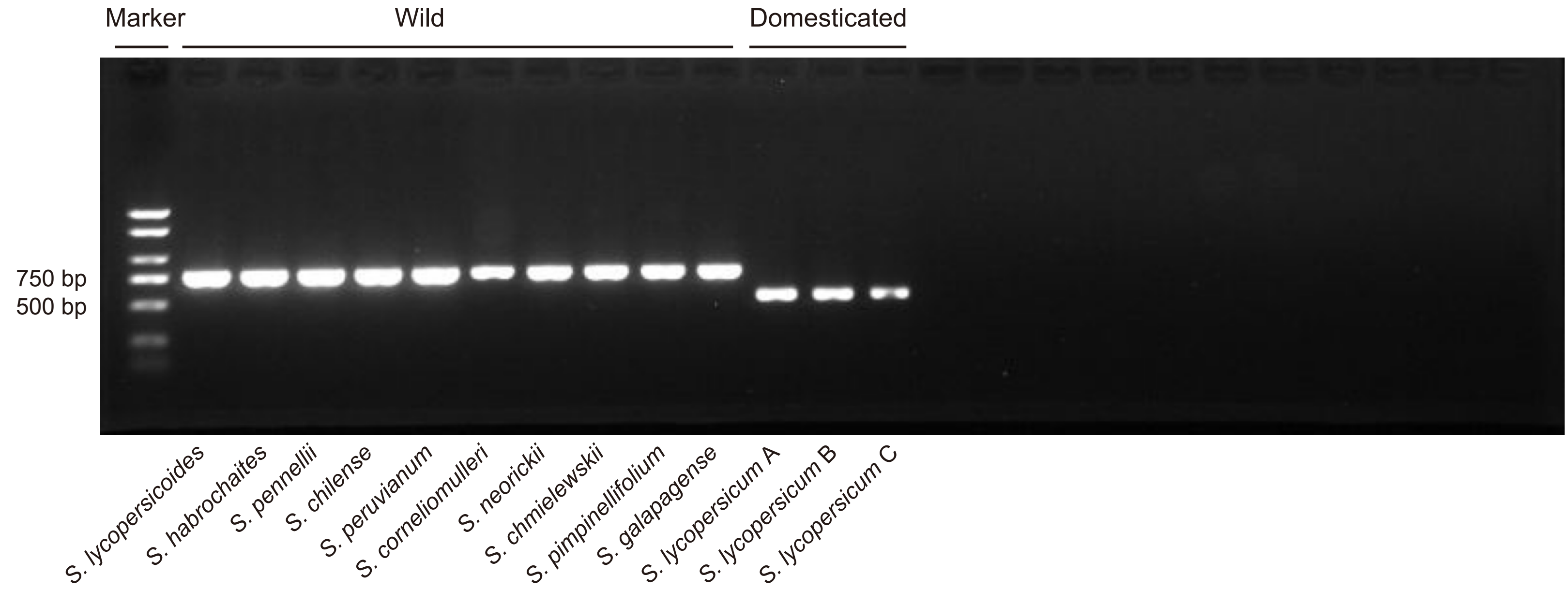

Supplement: Source Data Fig. 3 — Unprocessed gels for Fig. 3b. [file 41588_2023_1340_MOESM8_ESM.pdf]
